# Protein Poly(ADP-ribosyl)ation Regulates *Arabidopsis* Immune Gene Expression and Defense Responses

**DOI:** 10.1371/journal.pgen.1004936

**Published:** 2015-01-08

**Authors:** Baomin Feng, Chenglong Liu, Marcos V. V. de Oliveira, Aline C. Intorne, Bo Li, Kevin Babilonia, Gonçalo A. de Souza Filho, Libo Shan, Ping He

**Affiliations:** 1 Department of Biochemistry and Biophysics, and Institute for Plant Genomics & Biotechnology, Texas A&M University, College Station, Texas, United States of America; 2 Department of Plant Pathology and Microbiology, and Institute for Plant Genomics & Biotechnology, Texas A&M University, College Station, Texas, United States of America; 3 Center of Biosciences and Biotechnology, Darcy Ribeiro State University of Northern of Rio de Janeiro, Brazil; 4 Department of Biology, University of Puerto Rico, Mayagüez Campus, Mayagüez, Puerto Rico; Virginia Tech, United States of America

## Abstract

Perception of microbe-associated molecular patterns (MAMPs) elicits transcriptional reprogramming in hosts and activates defense to pathogen attacks. The molecular mechanisms underlying plant pattern-triggered immunity remain elusive. A genetic screen identified *Arabidopsis* poly(ADP-ribose) glycohydrolase 1 (*atparg1*) mutant with elevated immune gene expression upon multiple MAMP and pathogen treatments. Poly(ADP-ribose) glycohydrolase (PARG) is predicted to remove poly(ADP-ribose) polymers on acceptor proteins modified by poly(ADP-ribose) polymerases (PARPs) with three PARPs and two PARGs in *Arabidopsis* genome. AtPARP1 and AtPARP2 possess poly(ADP-ribose) polymerase activity, and the activity of AtPARP2 was enhanced by MAMP treatment. AtPARG1, but not AtPARG2, carries glycohydrolase activity *in vivo* and *in vitro*. Importantly, mutation (G450R) in *atparg1* blocks its activity and the corresponding residue is highly conserved and essential for human HsPARG activity. Consistently, mutant *atparp1atparp2* plants exhibited compromised immune gene activation and enhanced susceptibility to pathogen infections. Our study indicates that protein poly(ADP-ribosyl)ation plays critical roles in plant immune gene expression and defense to pathogen attacks.

## Author Summary

Fine-tuning of gene expression is a key feature of successful immune responses. However, the underlying mechanisms are not fully understood. Through a genetic screen in model plant *Arabidopsis*, we reveal that protein poly(ADP-ribosyl)ation (PARylation) post-translational modification plays a pivotal role in controlling plant immune gene expression and defense to pathogen attacks. PARylation is primarily mediated by poly(ADP-ribose) polymerase (PARP), which transfers ADP-ribose moieties from NAD^+^ to acceptor proteins. The covalently attached poly(ADP-ribose) polymers on the accept proteins could be hydrolyzed by poly(ADP-ribose) glycohydrolase (PARG). We further show that members of *Arabidopsis* PARPs and PARGs possess differential *in vivo* and *in vitro* enzymatic activities. Importantly, the *Arabidopsis parp* mutant displayed reduced, whereas *parg* mutant displayed enhanced, immune gene activation and immunity to pathogen infection. Moreover, *Arabidopsis* PARP2 activity is elevated upon pathogen signal perception. Compared to the lethality of their mammalian counterparts, the viability and normal growth of *Arabidopsis parp* and *parg* null mutants provide a unique genetic system to understand protein PARylation in diverse biological processes at the whole organism level.

## Introduction

Plants sense the presence of pathogens by the cell surface-localized pattern recognition receptors (PRRs), which perceive evolutionarily conserved pathogen- or microbe-associated molecular patterns (PAMPs or MAMPs), including bacterial flagellin, lipopolysaccharide (LPS), peptidoglycan (PGN), elongation factor Tu (EF-Tu), and fungal chitin [1]–[3]. A 22-amino-acid peptide corresponding to a region near the amino-terminus of flagellin (flg22) is recognized by the *Arabidopsis* PRR Flagellin-Sensing 2 (FLS2), a leucine-rich repeat receptor-like kinase (LRR-RLK) [4], [5]. Perception of flg22 by FLS2 induces instantaneous association with another LRR-RLK, Brassinosteroid Insensitive 1 (BRI1)-Associated Kinase 1 (BAK1), mainly through ectodomain heterodimerization of flg22-activated FLS2/BAK1 complex [6]–[9]. The receptor-like cytoplasmic kinases (RLCKs), BIK1 and its homolog PBL1, constitutively associate with FLS2 and BAK1, and are released from the receptor complex upon flg22 perception [10]–[12]. BAK1 directly interacts and phosphorylates BIK1 at both serine, threonine and tyrosine residues, thereby activating downstream signaling [12], [13]. In addition, both BAK1 and BIK1 complex with PRR EFR (receptor for EF-Tu) [11], [14], AtPEPR1 (receptor for endogenous danger signal Pep1) [12], [15], [16], and plant brassinosteroid hormone receptor BRI1 [17]–[19]. Activation of PRR complex by the corresponding MAMP triggers a series of defense responses, including rapid activation of MAP kinases (MAPKs) and calcium-dependent protein kinases, transient reactive oxygen species (ROS) production and calcium influx, stomatal closure, callose deposition and massive transcriptional reprogramming [1]–[3]. It has been shown recently that BIK1 is able to phosphorylate plasma membrane-resident NADPH oxidase family member respiratory burst oxidase homolog D (RBOHD), thereby contributing to ROS production [20], [21]. However, it remains largely unknown how PRR complex activation leads to profound immune gene transcriptional reprograming.

Protein poly(ADP-ribosyl)ation (PARylation), an important post-translational modification process, plays a crucial role in a broad array of cellular responses including DNA damage detection and repair, cell division and death, chromatin modification and gene transcriptional regulation [22]–[24] (S1 Fig.). PARylation is primarily mediated by members of poly(ADP-ribose) polymerases (PARPs), which transfer ADP-ribose moieties from nicotinamide adenine dinucleotide (NAD^+^) to different acceptor proteins at glutamate (Glu), aspartate (Asp) or lysine (Lys) residues resulting in the formation of linear or branched poly(ADP-ribose) (PAR) polymers on acceptor proteins (S1 Fig.). PAR activities and PARPs have been found in a wide variety of organisms from archaebacteria to mammals and plants, but they are apparently absent in yeast [25]. Human PARP-1 (HsPARP-1) is the most abundant and ubiquitous PARP among a family of 17 members, and it catalyzes the covalent attachment of PAR polymers on itself (auto-PARylation) and other target proteins, including histones, DNA repair proteins, transcription factors, and chromatin modulators [22]. HsPARP-1 possesses three functional domains with a DNA binding domain at N-terminus, auto-modification domain in the middle and a catalytic domain at C-terminus (S2A Fig.). PARylation is a reversible reaction and the covalently attached PAR on the target proteins can be hydrolyzed to free PAR or mono-(ADP-ribose) by poly (ADP-ribose) glycohydrolase (PARG) [22], [23] (S1 Fig.). PARG contains both endo- and exo-glycohydrolase activities that promote rapid catabolic destruction of PAR of target proteins [26]. There is only one *PARG* gene in humans with three different isoforms: PARG_99_ and PARG_102_ in the cytoplasm and PARG_110_ in the nucleus [26]. Mammalian PARG possesses a regulatory and targeting domain (A-domain) at the N-terminus, a mitochondrial targeting sequence (MTS) in the middle and a conserved catalytic domain at the C-terminus [27] (S2B Fig.). The catalytic core containing “GGG-X_6-8_-QEE” PARG signature motif interacts with PAR and executes hydrolysis activity [28]. Despite of their apparently opposing activities, members of PARPs and PARGs coordinately regulate protein PARylation and play essential roles in a wide range of cellular processes and contribute to the pathogenicity of various diseases, including cancer, cardiovascular diseases, stroke, metabolic disorders, diabetes and autoimmunity [25].

The *Arabidopsis* genome encodes three members of PARPs, *AtPARP1* (*At2g31320*), *AtPARP2* (*At4g02390*) and *AtPARP3* (*At5g22470*) and two members of PARGs, *AtPARG1* (*At2g31870*) and *AtPARG2* (*At2g31865*) [23], [29] (S2 Fig.). AtPARP1 (it was originally named as AtPARP2) shares the conserved domain structure with HsPARP-1, whereas AtPARP2 (it was originally named as AtPARP1) and AtPARP3 more closely resemble HsPARP-2 and HsPARP-3 [29] (S2A Fig.). As their mammalian counterparts, plant PARPs are implicated in DNA repair, cell cycle and genotoxic stress [29]-[32]. Importantly, plant PARPs play an essential role in response to abiotic stresses. Transgenic *Arabidopsis* or oilseed rape (*Brassica napus*) plants with reduced *PARP* gene expression were more resistant to various abiotic stresses, including drought, high light and heat, partially attributed to a maintained energy homeostasis of reduced NAD^+^ and ATP consumption and alternation in plant hormone abscisic acid (ABA) levels in the transgenic plants [33], [34]. The two *Arabidopsis* PARG genes, *AtPARG1* and *AtPARG2*, which were likely derived from a tandem duplication event, locates next to each other on the same chromosome [23]. AtPARG1 (TEJ) was originally identified as a regulator of circadian rhythm and flowering in *Arabidopsis* [35]. Interestingly, the *AtPARG2* gene was robustly induced by the treatments of MAMPs and various pathogens [36]. The plants carrying mutation in *AtPARG1*, but not *AtPARG2*, showed the elevated elf18 (a 18-amino-acid peptide of EF-Tu)-mediated seedling growth inhibition and phenylpropanoid pigment accumulation, suggesting a negative role of *Arabidopsis* PARG in certain plant immune responses [37]. Similar to AtPARP1, AtPARG1 also plays a role in plant drought, osmotic and oxidative stress tolerance [38]. In contrast to the extensive research efforts on PARPs/PARGs in animal systems, the biochemical activities and molecular actions of plant PARPs/PARGs remain poorly characterized.

To elucidate the signaling networks regulating immune gene activation, we developed a sensitive genetic screen with an ethyl methanesulfonate (EMS)-mutagenized population of *Arabidopsis* transgenic plants carrying a luciferase reporter gene under the control of the *FRK1* promoter (*pFRK1::LUC*). The *FRK1* (flg22-induced receptor-like kinase 1) gene is a specific and early immune responsive gene activated by multiple MAMPs [39], [40]. A series of mutants with altered *pFRK1::LUC* activity upon flg22 treatment were identified and named as *Arabidopsis* genes governing immune gene expression (*aggie*). In this study, we isolated and characterized the *aggie2* mutant, which exhibited elevated immune gene expression upon multiple MAMP treatments. Map-based cloning coupled with next generation sequencing revealed that *Aggie2* encodes AtPARG1. Extensive biochemical analysis demonstrates that both AtPARP1 and AtPARP2 carry poly(ADP-ribose) polymerase activity, whereas AtPARG1, but not AtPARG2, possesses poly(ADP-ribose) glycohydrolase activity *in vivo* and *in vitro*. Significantly, the enzymatic activity of AtPARP2 is enhanced upon flg22 perception, suggesting the potential involvement of protein PARylation in MAMP-triggered immunity. The *aggie2* mutation (G450R) occurs at a highly conserved PARG residue which is essential for both *Arabidopsis* AtPARG1 and human HsPARG enzymatic activity. Consistent with the negative role of AtPARG1 in plant innate immunity, AtPARP1 and AtPARP2 positively regulate immune gene activation and plant resistance to virulent bacterial pathogen infection. Our results indicate that the reversible posttranslational PARylation process mediated by AtPARPs and AtPARGs plays a crucial role in mounting successful innate immune responses upon MAMP perception in *Arabidopsis*.

## Results

### The *aggie2* mutant displays enhanced immune gene expression

**Figure 1:**
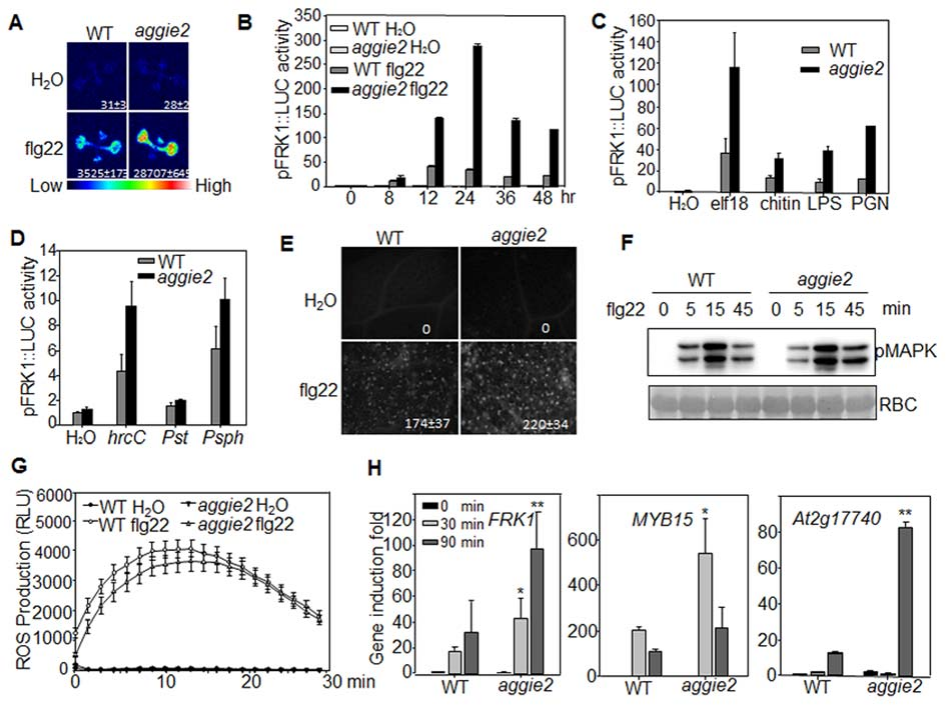
Elevated *pFRK1::LUC* expression and MAMP-triggered immune response in *aggie2* mutant. (**A**) Luciferase activity from 10-day-old *pFRK1::LUC* (WT) and *aggie2* seedlings treated with or without 10 nM flg22 for 12 hr. The photograph was taken with an EMCCD camera. The number below indicates quantified signal intensity shown as means ± se from 12 seedlings. (**B**) Time-course of *pFRK1::LUC* activity in response to 100 nM flg22 treatment. The data are shown as means ± se from at least 20 seedlings for each time point. (**C**) The *pFRK1::LUC* activity in response to different MAMPs. Ten-day-old seedlings were treated with 100 nM elf18, 50 µg/ml chitin, 1 µM LPS, or 500 ng/ml PGN for 12 hr. The data are shown as means ± se from at least 12 seedlings for each treatment. (**D**) The *pFRK1::LUC* activity triggered by different bacteria. Four-week-old soil-grown plants were hand-inoculated with different bacteria at the concentration of OD_600_  =  0.5. The data are shown as means ± se from at least 12 leaves for each treatment at 24 hr post-inoculation (hpi). (**E**) flg22-induced callose deposition in *aggie2* mutant. Leaves of 6-week-old plants were infiltrated with 0.5 µM flg22 for 12 hr and callose deposits were detected by aniline blue staining and quantified by ImageJ software. (**F**) flg22-induced MAPK activation in *aggie2* mutant. Seedlings were treated with 100 nM flg22 and collected at the indicated time points. The MAPK activation was detected with an α-pErk antibody (top panel) and the protein loading was indicated by Ponceau S staining for RuBisCo (RBC) (bottom panel). (**G**) flg22-triggered ROS burst in *aggie2* mutant. Leave discs from 4-week-old plants were treated with H_2_O or 100 nM flg22 over 30 min. The data are shown as means ± se from 20 leaf discs. (**H**) Endogenous MAMP-induced marker gene expression. Ten-day-old seedlings were treated with 100 nM flg22 for 30 and 90 min for qRT-PCR analysis. The data are shown as means ± se from three biological repeats with Student's *t*-test. * indicates p<0.05 and ** indicates p<0.01 when compared to WT. The above experiments were repeated 3 times with similar results.

The *aggie2* mutant isolated from a genetic screen of the EMS-mutagenized *pFRK1::LUC* transgenic plants exhibits elevated *FRK1* promoter activity upon flg22 treatment compared to its parental line, *pFRK1::LUC* (WT) (Fig. 1A). The elevated luciferase activity in the *aggie2* mutant was observed over a 48-hr time course period upon flg22 treatment (Fig. 1B). Notably, the *aggie2* mutant did not display detectable enhanced *FRK1* promoter activity in the absence of flg22 treatment, suggesting its specific regulation in plant defense. In addition to flg22, other MAMPs, including elf18, LPS, PGN and fungal chitin, also elicited the enhanced *FRK1* promoter activity in the *aggie2* mutant (Fig. 1C), indicating that Aggie2 functions as a convergent component downstream of multiple MAMP receptors. Consistently, the *aggie2* mutant displayed the enhanced *FRK1* promoter activity in response to the non-pathogenic bacterium *Pseudomonas syringae* pv. *tomato* (*Pst*) DC3000 *hrcC* defective in type III secretion of effectors, and a non-adaptive bacterium *P. syringae* pv. *phaseolicola* NPS3121 (Fig. 1D). The pathogenic bacterium *Pst* DC3000 failed to activate *pFRK1::LUC*, likely due to the suppression function of multiple effectors secreted from virulent bacterium [40]. Pathogen infection or purified MAMPs could induce callose deposits in leaves or cotyledons of *Arabidopsis*, which has emerged as an indicator of plant immune responses [41]. We compared callose deposits by aniline blue staining in WT and *aggie2* mutant plants upon flg22 treatment. The *aggie2* mutant deposited more callose than WT plants 12 hr after flg22 treatment, and the size of each callose deposit appeared bigger in the *aggie2* mutant than that in WT plants (Fig. 1E).

We also detected MAPK activation and ROS production, two early events triggered by multiple MAMPs, in WT and *aggie2* mutant. The flg22-induced MAPK activation detected by an α-pERK antibody did not show significant and reproducible difference in WT and *aggie2* seedlings (Fig. 1F), suggesting that *Aggie2* acts either independently or downstream of MAPK cascade. The flg22-induced ROS burst appeared to be similar in the *aggie2* mutant compared to that in WT plants (Fig. 1G). We did not observe reproducible disease alternation in the *aggie2* mutant compared to WT plants in response to *Pst* DC3000 infection either by hand-infiltration or spray-inoculation with various inoculums and conditions (S3A Fig.). Among 7 times of disease assays with *Pst* DC3000 hand-infiltration, we observed that *aggie2* was slightly more resistant than WT plants for 4 times, whereas we did not see the significant difference between *aggie2* and WT for other 3 times (S3A Fig). By contrast, the *aggie2* mutant showed enhanced susceptibility to a necrotrophic fungus *Botrytis cinerea* compared to WT plants as evidenced by symptom development and lesion progression after infection (S3B Fig).

We further detected endogenous *FRK1* expression in flg22-treated seedlings of WT and *aggie2* mutant with quantitative reverse transcription-polymerase chain reaction (qRT-PCR) analysis. The *FRK1* expression was significantly elevated in the *aggie2* mutant compared to that of WT *pFRK1::LUC* transgenic plants at both 30 min and 90 min after flg22 treatment (Fig. 1H). Similarly, the expression of several other early MAMP marker genes, including *MYB15* and *At2g17740* was also enhanced in the *aggie2* mutant (Fig. 1H). Taken together, the results indicate that Aggie2 negatively regulates the expression of certain flg22-induced genes.

### *Aggie2* encodes a putative poly(ADP-ribose) glycohydrolase

**Figure 2:**
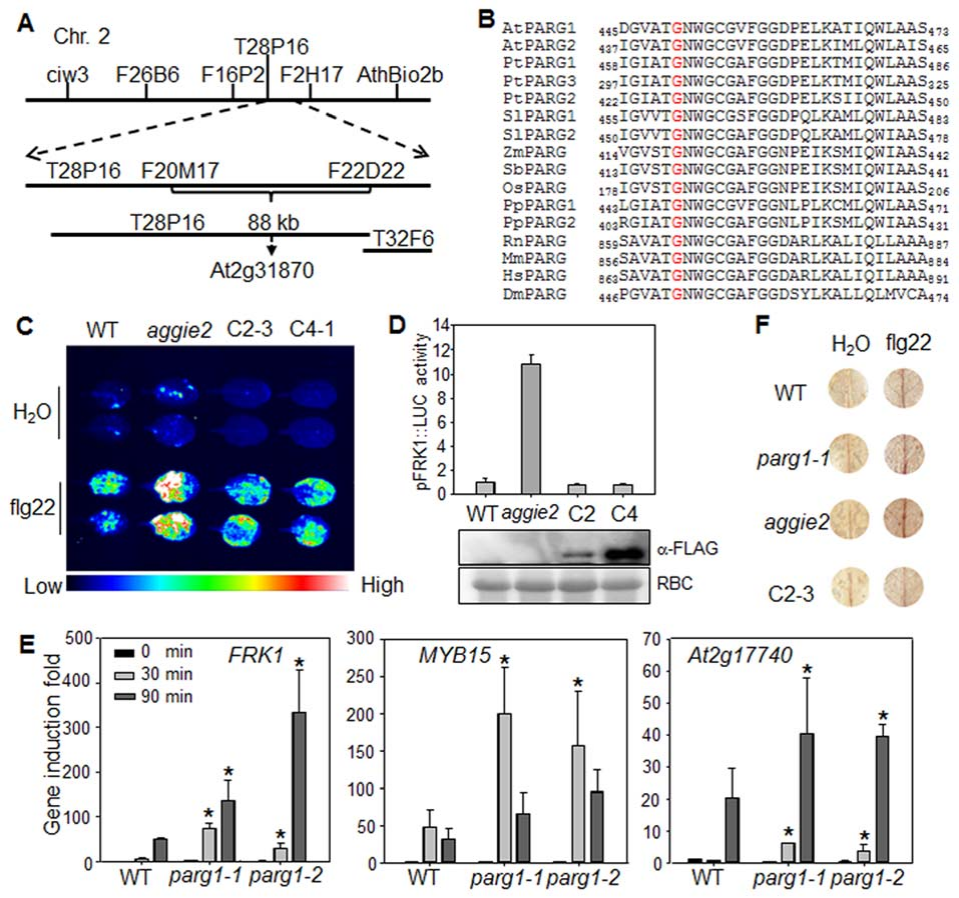
*Aggie2* encodes AtPARG1. (**A**) Mapping of *aggie2* on chromosome 2 and NGS identified a G to A mutation in *At2g31870*, which encodes AtPARG1. (**B**) G450 (red) in AtPARG1, which was mutated to Arginine (R) in *aggie2*, is highly conserved among different PARGs from plants to animals, thale cress (*Arabidopsis thaliana*, At), poplar (*Populus trichocarpa*, Pt), tomato (*Solanum lycopersicum*, Sl), maize (Zea mays, Zm), sorghum (*Sorghum bicolor*, Sb), rice (*Oryza sativa*, Os), moss (*Physcomitrella patens*, Pp), rat (*Rattus norvegicus*, Rn), mouse (*Mus musculus*, Mm), human (*Homo sapiens*, Hs), fruit fly (*Drosophila melanogaster*, Dm). (**C**) and (**D**) AtPARG1 complements *aggie2* mutant phenotype. Leaves of 4-week-old soil-grown plants were treated with or without 100 nM flg22 for 12 hr and photographed with an EMCCD camera (**C**) and quantification data are shown in (**D**). The *pFRK1::LUC* transgenic plants (WT), *aggie2* mutant and two independent homozygous lines, 2-3 and 4-1, of *pAtPARG1::AtPARG1-FLAG* transgenic plants in *aggie2* mutant background were included in the assay. The data are shown as means ± se from at least 10 seedlings. The protein expression of the transgene was detected with an α-FLAG Western blot. (**E**) Enhanced immune gene expression in *atparg1* mutant. Ten-day-old seedlings were treated with 100 nM flg22 for 30 and 90 min for qRT-PCR analysis. The data are shown as means ± se from three biological repeats with Student's *t*-test. * indicates p<0.05 when compared to WT. (**F**) flg22-induced lignin biosynthesis precursors in *aggie2* mutant. Leaves of 6-week-old plants were incubated with 100 nM flg22 for 12 hr and lignin precursors, O-4-linked-coniferyl and sinapyl aldehydes, were detected by Wiesner staining. The images were scanned by HP officejet Pro 8600 Premium. The above experiments were repeated 3 times with similar results.

To isolate the causative mutation in *aggie2*, we crossed *aggie2* (in the Col-0 accession background) with the *Ler* accession and mapped *aggie2* to an 88 kilobase pair (kb) region between markers F20M17 and F22D22 on Chromosome 2 (Fig. 2A). We then performed Illumina whole genome sequencing of *aggie2* and WT *pFRK1::LUC* transgenic plants. The comparative sequence analysis identified a G to A mutation at the position 1348 bp of *At2g31870* within this 88 kb region. The mutation was further confirmed by Sanger sequencing of the genomic DNA of *At2g31870*. *At2g31870* encodes AtPARG1 and the mutation in the *aggie2* mutant causes an amino acid change of Glycine (G) at 450 to Arginine (R) (G450R) (Fig. 2B). The G450 in AtPARG1 resides in a highly conserved region at the C-terminus with unknown function. Notably, this residue is invariable in different species of plants and animals, including *Arabidopsis*, poplar, tomato, maize, sorghum, rice, moss, rat, mouse, human and fruit fly, suggesting the essential role of this residue in PARG functions (Fig. 2B).

To confirm that the G450R lesion in AtPARG1 is the causative mutation in *aggie2*, we complemented the *aggie2* mutant with a construct carrying *AtPARG1* cDNA fused with a FLAG epitope tag under the control of its native promoter (*pAtPARG1::AtPARG1-FLAG*). Two homozygous T3 transgenic lines, one line with relatively low (C2-3) and another line with moderate (C4-1) expression of AtPARG1-FLAG, were chosen for complementation assays. Both lines restored WT level of *pFRK1::LUC* activity upon flg22 treatment either imaged with an EMCCD camera (Fig. 2C) or quantified by a luminometer (Fig. 2D), confirming that the enhanced *FRK1* promoter activity in *aggie2* is caused by the mutation in *AtPARG1*. We also isolated T-DNA insertion line of *AtPARG1, parg1-1* (*SALK_147805*) and *parg1-2* (*SALK_116088*), and examined flg22-induced immune gene activation. Similar to the *aggie2* mutant, *parg1-1* and *parg1-2* displayed the elevated activation of *FRK1*, *MYB15* and *At2g17740* after flg22 treatment compared to WT Col-0 plants (Fig. 2E). PARP inhibitor disrupted MAMP-induced cell wall lignification [37]. We found that both *parg1-1* and *aggie2* mutants showed the enhanced accumulation of lignin biosynthesis precursors, O-4-linked-coniferyl and sinapyl aldehydes, upon flg22 treatment by Wiesner staining (Fig. 2F). The complementation line C2-3 restored accumulation of these lignin biosynthesis precursors to the WT level (Fig. 2F). Consistent with a previous report [36], the transcript of *AtPARG2*, but not *AtPARG1*, was induced by flg22 treatment (S4A Fig.).

### AtPARP1 and AtPARP2 carry poly(ADP-ribose) polymerase activity *in vitro*

AtPARG1 encodes a putative poly(ADP-ribose) glycohydrolase with a predicated activity to remove poly(ADP-ribose) polymers on the acceptor proteins catalyzed by poly(ADP-ribose) polymerases (PARPs). To elucidate the biochemical activity and function of AtPARGs, we first characterized the function of AtPARPs and established *in vivo* and *in vitro* protein PARylation assays. The *Arabidopsis* genome encodes three PARPs, AtPARP1, AtPARP2 and AtPARP3, with each consisting of a conserved PARP catalytic domain and a variable DNA binding domain (S2 Fig.). AtPARP1 and AtPARP3 carry zinc-finger domains for DNA binding, which is similar with human HsPARP-1, whereas AtPARP2 contains two SAP domains with putative DNA binding activity. The SAP domain was named after scaffold attachment factor A/B (SAF-A/B), apoptotic chromatin condensation inducer in the nucleus (Acinus) and protein inhibitors of activated STAT (PIAS), which all have DNA and chromatin binding ability and regulate chromatin structure and/or transcription [42]. Analysis of their tissue expression pattern suggests that *AtPARP1* and *AtPARP2* are expressed in leaves, whereas *AtPARP3* is primarily expressed in developing seeds (S4B-S4C Fig.). Thus, we focused on AtPARP1 and AtPARP2 for the functional studies.

**Figure 3:**
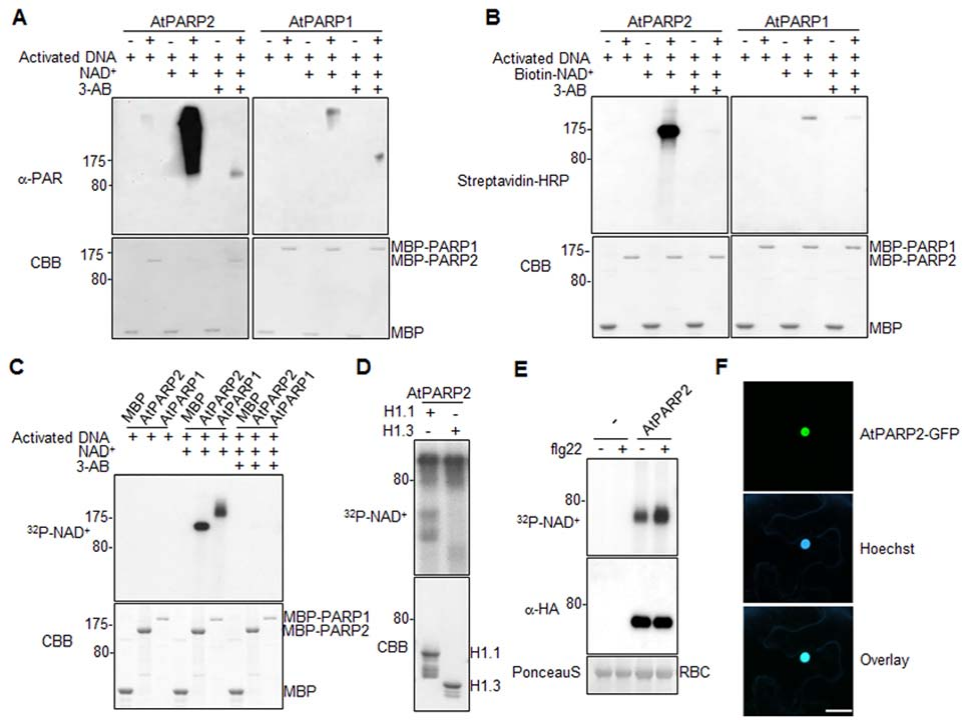
*Arabidopsis* AtPARP1 and AtPARP2 are functional poly(ADP-ribose) polymerases. (**A**) AtPARP1 and AtPARP2 *in vitro* activity detected by α-PAR antibody. MBP, MBP-AtPARP1 or MBP-AtPARP2 proteins were incubated with activated DNA with or without NAD^+^. 3-AB is a competitive inhibitor of PARP and could block PAR reactions. The PARylated proteins were detected in a Western blot using α-PAR antibody (top Panel) and the protein inputs were indicated by Coomassie Brilliant Blue (CBB) staining (bottom panel). The blot exposure time for AtPARP1 and AtPARP2 was same. (**B**) AtPARP1 and AtPARP2 *in vitro* activity detected by Biotin-labelled NAD^+^. (**C**) AtPARP1 and AtPARP2 *in vitro* activity detected by autoradiograph with ^32^P-NAD^+^. (**D**) AtPARP2 PARylates *Arabidopsis* Histone H1.1 and H1.3 *in vitro*. The PARylated proteins were detected by autoradiography with ^32^P-NAD^+^. (**E**) flg22 treatment enhances AtPARP2 activity *in vivo*. Protoplasts were transfected with *AtPARP2-HA*, treated with 100 nM flg22 for 30 min and fed with ^32^P-NAD^+^. The AtPARP2-HA proteins were immunoprecipitated with an α-HA antibody and detected by autoradiography (Top panel). The input of AtPARP2 proteins is shown with an α-HA Western blot (middle panel), and the protein loading control is shown by Ponceau S staining for RBC (bottom panel). (**F**) AtPARP2 localizes in nucleus. *AtPARP2-GFP* was transiently expressed in *N. benthamiana* and the images were taken with a confocal microscope 2 days after inoculation. For nuclear staining, Hoechst 33342 (1 µg/ml) was infiltrated into the *N. benthamiana* leaf one hour before imaging. Scale bar is 20 µm. The above experiments were repeated at least 3 times with similar results.

We first tested whether AtPARP1 and AtPARP2 carry poly(ADP-ribose) polymerase activity with recombinant proteins of AtPARP1 and AtPARP2 fused with Maltose Binding Protein (MBP). In the presence of activated DNA, both AtPARP1 and AtPARP2 could catalyze PARylation reaction by repeatedly transferring ADP-ribose groups from NAD^+^ to itself (auto-PARylation) as appeared a ladder-like smear with high-molecular-weight proteins in a Western blot using an α-PAR antibody which detects the PAR polymers of PARylated proteins (Fig. 3A). Apparently, AtPARP2 exhibited stronger *in vitro* enzymatic activity than AtPARP1 when detected by α-PAR antibody. The enzymatic activity of AtPARP1 and AtPARP2 was blocked by 3-AB, a competitive inhibitor of PARP (Fig. 3A). The activity of AtPARP2 is comparable with that of human HsPARP-1 (S5A Fig.). In addition, both AtPARP1 and AtPARP2 were able to transfer ADP-ribose from Biotin-NAD^+^ to itself and a relatively discrete band could be detected by horseradish peroxidase (HRP) conjugated streptavidin (Fig. 3B). The specificity of PARP activity was confirmed with 3-AB treatment, which dramatically reduced auto-PARylation. Similar with the observation using α-PAR antibody, AtPARP2 exhibited stronger *in vitro* enzymatic activity than AtPARP1 when detected by streptavidin-HRP for biotinylated NAD^+^. We further developed a PARylation assay with radiolabeled ^32^P-NAD^+^ as the ADP-ribose donor (Fig. 3C). Clearly, both AtPARP1 and AtPARP2 were able to transfer ADP-ribose from ^32^P-NAD^+^ to itself as shown with SDS-PAGE autoradiograph (Fig. 3C). The formation of relatively discrete band was likely caused by these assay conditions, which favor synthesis of short polymers due to limited amount of NAD^+^ [43]. Together, the data support that both AtPARP1 and AtPARP2 are active poly(ADP-ribose) polymerases *in vitro*. It has been shown that human HsPARP-1 could modify linker histone H1 proteins and thereby create a chromatin structure more accessible to RNA polymerase II (RNAPII) to regulate transcription [44]. We further examined whether AtPARP2 was also able to PARylate *Arabidopsis* histone proteins. As detected with radiolabeled ^32^P-NAD^+^, AtPARP2 could PARylate two *Arabidopsis* histone proteins H1.1 and H1.3 (Fig. 3D). It is possible that *Arabidopsis* PARPs may use a similar mechanism for transcriptional regulation.

### Flg22 induces AtPARP2 activity *in vivo*

We further developed an *in vivo* PARylation assay with transiently expressed AtPARP2 tagged with an HA epitope at the C-terminus in *Arabidopsis* protoplasts. After feeding the cells with ^32^P-NAD^+^, the AtPARP2 proteins were immunoprecipitated with an α-HA antibody and separated in SDS-PAGE. A band corresponding to the predicated molecular weight of AtPARP2 was observed with autoradiograph, indicating *in vivo* AtPARP2 activity (Fig. 3E). This band is specific to AtPARP2 since it was absent in the vector control transfected cells. Strikingly, the flg22 treatment enhanced AtPARP2 *in vivo* PARylation activity as detected by increased band intensity with autoradiograph. Apparently, the flg22-mediated enhancement of AtPARP2 activity was not due to the increase of protein expression after treatment (Fig. 3E). The data demonstrate that AtPARP2 possesses poly(ADP-ribose) polymerase activity *in vivo* and AtPARP2-mediated protein PARylation is regulated by flg22 signaling. We further examined AtPARP2-GFP localization with *Agrobacterium*-mediated *Nicotiana benthamiana* transient assay. A strong fluorescence signal from AtPARP2-GFP was exclusively detected in the nucleus (Fig. 3F), which is consistent with its potential role in DNA repair, chromatin modulation and transcriptional regulation.

### AtPARG1, but not AtPARG2, is a functional PARG enzyme

**Figure 4:**
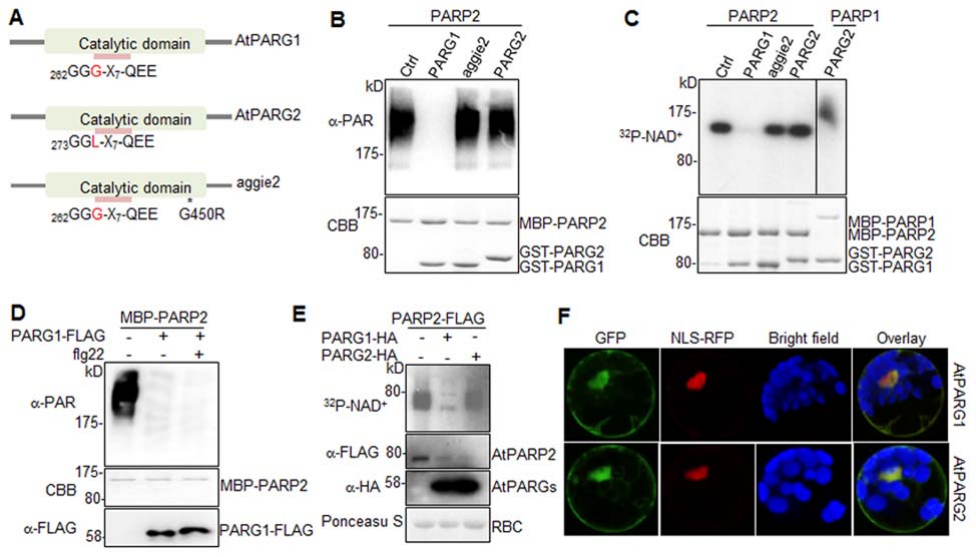
AtPARG1, but not aggie2 or AtPARG2, has poly(ADP-ribose) glycohydrolase activity. (**A**) Schematic catalytic domain of AtPARG1, AtPARG2 and aggie2. The sequence of the PARG signature motif is shown and the number indicates the position of the first glycine (G) residue with a polymorphic residue G and L (leucine) between AtPARG1 and AtPARG2 highlighted in red. * denotes the point mutation in aggie2. (**B**) AtPARG1, but not AtPARG2 nor aggie2, possesses *in vitro* PAR glycohydrolase activity towards auto-PARylated AtPARP2 proteins detected by α-PAR antibody. MBP-AtPARP2 proteins were auto-PARylated and further subjected for *in vitro* PARG assay using GST-tagged AtPARG1, aggie2 or AtPARG2 proteins. The PARylated proteins were detected with an α-PAR Western blot (top panel) and the protein inputs are shown with CBB staining (bottom panel). (**C**) The AtPARG *in vitro* activity detected with ^32^P-NAD^+^. AtPARG1, but not AtPARG2 or aggie2, possesses *in vitro* PARG activity towards auto-PARylated AtPARP2 proteins (left part of top panel) and AtPARG2 does not have PARG activity towards auto-PARylated AtPARP1 proteins (right part of top panel). MBP-AtPARP1 or AtPARP2 proteins were auto-PARylated and further subjected for *in vitro* PARG assays using GST-tagged PARG1, aggie2 or PARG2 proteins in the presence of ^32^P-NAD^+^. The PARylated proteins were detected with autoradiograph (top panel) and the protein inputs are shown with CBB staining (bottom panel). (**D**) Protoplast-expressed AtPARG1 possesses PARG activity towards *in vitro* auto-PARylated AtPARP2 proteins. *Arabidopsis* protoplasts were transfected with *AtPARG1-FLAG* or vector control and treated with or without 100 nM flg22 for 15 min. PARG1 proteins were immunoprecipitated with α-FLAG antibody and subjected for *in vitro* PARG assay with *in vitro* auto-PARylated MBP-AtPARP2 proteins. The PARylated proteins were detected in an α-PAR Western blot (top panel), MBP-AtPARP2 protein input is shown with CBB staining (middle panel) and AtPARG1-FLAG protein expression in protoplasts is shown with an α-FLAG Western blot (bottom panel). (**E**) AtPARG1, but not AtPARG2, has *in vivo* PAR glycohydrolase activity. AtPARP2-FLAG was co-expressed with vector control, AtPARG1-HA or AtPARG2-HA in protoplasts and, the protoplasts were fed with ^32^P-NAD^+^. The PARylated proteins were detected with autoradiograph after immunoprecipitation with α-FLAG antibody (top panel). The PARP and PARG protein expression was detected with Western blot (middle panels) and the protein loading is shown with Ponceau S staining (bottom panel). (**F**) Subcellular localization of AtPARG1 and AtPARG2 in protoplasts. AtPARG1-GFP or AtPARG2-GFP was transiently expressed in protoplasts and the images were taken 12 hr after transfection using a confocal microscope. NLS-RFP was co-transfected for nuclear localization control. The above experiments were repeated 3 times with similar results.

We next tested whether AtPARG1 and AtPARG2 possess poly(ADP-ribose) glycohydrolase activity (Fig. 4A). We isolated and purified AtPARG1 and AtPARG2 proteins fused with glutathione S-transferase (GST) expressed from *E. coli*, and established an *in vitro* PARG assays to examine whether AtPARGs could remove PAR from auto-PARylated AtPARP2 *in vitro*. As shown in Fig. 4B, AtPARG1 diminished the formation of the ladder-like smear of auto-PARylated AtPARP2 detected in a Western blot with an α-PAR antibody, suggesting the PARG activity of AtPARG1 towards AtPARP2. However, AtPARG2 appeared to be inactive towards auto-PARylated AtPARP2 in this assay (Fig. 4B). Similarly, AtPARG1, but not AtPARG2, could remove PAR polymers from auto-ADP-ribosylated AtPARP2 as detected with ^32^P-NAD^+^ autoradiograph (Fig. 4C). We further examined whether AtPARG2 may possess PARG activity specifically towards AtPARP1 but not AtPARP2. As shown in Fig. 4C, AtPARG2 did not remove PAR polymers from auto-ADP-ribosylated AtPARP1. The 6xHistidine (His6)-tagged AtPARG2 also did not display *in vitro* enzymatic activity (S5B Fig.). Similar to the above assays using *in vitro* expressed AtPARG1 proteins (Fig. 4B & 4C), the immunoprecipitated AtPARG1 expressed in *Arabidopsis* protoplasts almost completely removed PAR polymers from *in vitro* PARylated AtPARP2 (Fig. 4D). Furthermore, AtPARG1 was able to remove PAR polymers from auto-PARylated human HsPARP-1 (S5C Fig.). Similarly, human HsPARG was also able to remove PAR polymers from AtPARP2 (S5D Fig.), suggesting the functional conservation of human and *Arabidopsis* PARPs/PARGs.

To test whether AtPARGs carry enzymatic activity *in vivo*, HA-tagged AtPARG1 or AtPARG2 was co-expressed with FLAG-tagged AtPARP2 transiently expressed in *Arabidopsis* protoplasts. After feeding the protoplasts with ^32^P-NAD^+^, AtPARP2 activity was detected with autoradiograph after immunoprecipitation with an α-FLAG antibody. Significantly, co-expression of AtPARG1, but not AtPARG2, substantially removed PAR polymers from *in vivo* PARylated AtPARP2 (Fig. 4E). The expression level of AtPARG1 and AtPARG2 was similar in protoplasts as detected by an α-HA Western blot (Fig. 4E). Taken together, our data indicate that AtPARG1 has *in vivo* and *in vitro* poly(ADP-ribose) glycohydrolase activity and AtPARG2 activity was not detected. Consistently, the *parg1* mutant, but not *parg2* mutant, accumulated higher PAR polymers than Col-0 with dot blotting of nuclear proteins by α-PAR antibody (S5E Fig.). Subcellular localization study indicates that AtPARG1-GFP and AtPARG2-GFP reside mainly in nucleus, but also in plasma membrane and cytoplasm when transiently expressed in *Arabidopsis* protoplasts (Fig. 4F).

**Figure 5:**
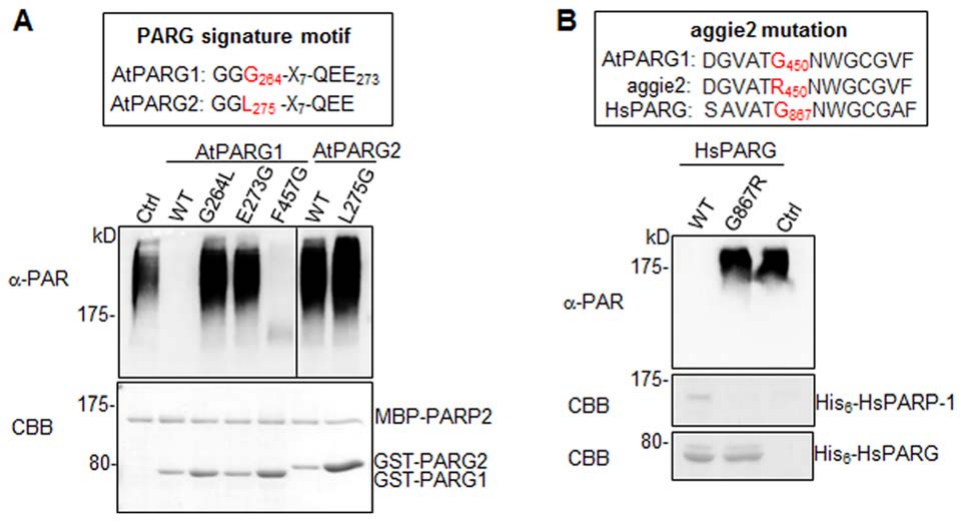
The signature motif and residue G450 are essential for PARG enzymatic activity. (**A**) The conserved residues G264 and E273 in the PARG signature motif of AtPARG1 are required for its enzymatic activity (left part of top panel) and creating a conserved PARG signature motif in AtPARG2 did not make it enzymatically active (right part of top panel). The sequences of PARG signature motif in AtPARG1 and AtPARG2 are shown on the top with the polymorphic residue labeled in red. F457 locates outside of the PARG signature motif in AtPARG1 and is not required for its enzymatic activity. AtPARP2 proteins were auto-PARylated and further subjected for PARG assay with WT AtPARG1, AtPARG2 or different mutated variants of AtPARGs. The PARylated proteins were detected with an α-PAR Western blot (top panel) and protein inputs are shown with CBB staining (bottom panel). (**B**) G450 in AtPARG1 is an essential residue in human HsPARG. Alignment of sequences of AtPARG1, aggie2 and HsPARG around AtPARG1G450 (red) residue is shown on the top. The corresponding G867R mutation in HsPARG blocked its activity to hydrolyze auto-PARylated human HsPARP-1. The auto-PARylated human HsPARP-1 proteins were incubated with WT or mutant form of HsPARG for PARG assay. The PARylated proteins were detected with an α-PAR Western blot (top panel) and protein inputs are shown with CBB staining (middle & bottom panels). The above experiments were repeated 3 times with similar results.

Notably, AtPARG1 protein possesses a PARG signature motif with the conserved sequence of “GGG-X_7_-QEE”. Mutation of E273 (the last E in the signature motif) in AtPARG1 to glycine (E273G) blocked its enzymatic activity, implicating the importance of this signature motif in PARG enzymatic activity (Fig. 5A). Examination of AtPARG2 sequence revealed that AtPARG2 has a polymorphism in the PARG signature motif. Instead of the conserved sequence “GGG-X_7_-QEE”, AtPARG2 possesses “GGL-X_7_-QEE” (Fig. 4A and 5A). The importance of this residue was shown by that the mutation of G264 (third G in the signature motif) in AtPARG1 to leucine (G264L) blocked its PARG enzymatic activity (Fig. 5A). We further determined whether lack of enzymatic activity of AtPARG2 (Fig. 4B, 4C & 4E) is due to this polymorphism in the PARG signature motif. We mutated leucine (L275) in AtPARG2 to glycine and generated the conserved “GGG-X_7_-QEE” motif. However, AtPARG2L275G mutant with a perfectly conserved PARG signature motif still did not show any detectable poly(ADP-ribose) glycohydrolase activity (Fig. 5A). The data suggest that the polymorphism of the PARG signature motif in AtPARG2 is not the sole determinant of its lack of detectable enzymatic activity and additional polymorphisms/deletions also account for its loss of PARG functions. There are only about 52% amino acid identity and 66% similarity between AtPARG1 and AtPARG2 (S6 Fig.).

### The *aggie2* mutation occurs at a conserved and essential PARG residue

We further addressed whether the aggie2 (G450R) mutation affected its PARG activity. Significantly, the aggie2 (G450R) mutant of AtPARG1 completely abolished its enzymatic activity detected by either α-PAR antibody (Fig. 4B) or ^32^P-NAD^+^ autoradiograph-based assay (Fig. 4C). Notably, the G450 in AtPARG1 is highly conserved among PARGs of different species (Fig. 2B). Interestingly, the corresponding mutation in human HsPARG (G867R) also abolished its activity towards HsPARP-1 and AtPARP2, suggesting the essential role of this highly conserved residue in different PARGs (Fig. 5B and S5D Fig.). The phenylalanine (F) at position 227 in bacterium *Thermomonospora curvata* PARG is implicated in positioning the terminal ribose and the mutation of which rendered the enzyme inactive [28]. Surprisingly, mutation of the corresponding residue F457 to glycine (F457G) in AtPARG1 did not affect its enzymatic activity (Fig. 5A), suggesting a possible distinct function mediated by this residue in different PARGs and potentially divergent evolution.

### AtPARPs positively regulate plant immunity

**Figure 6:**
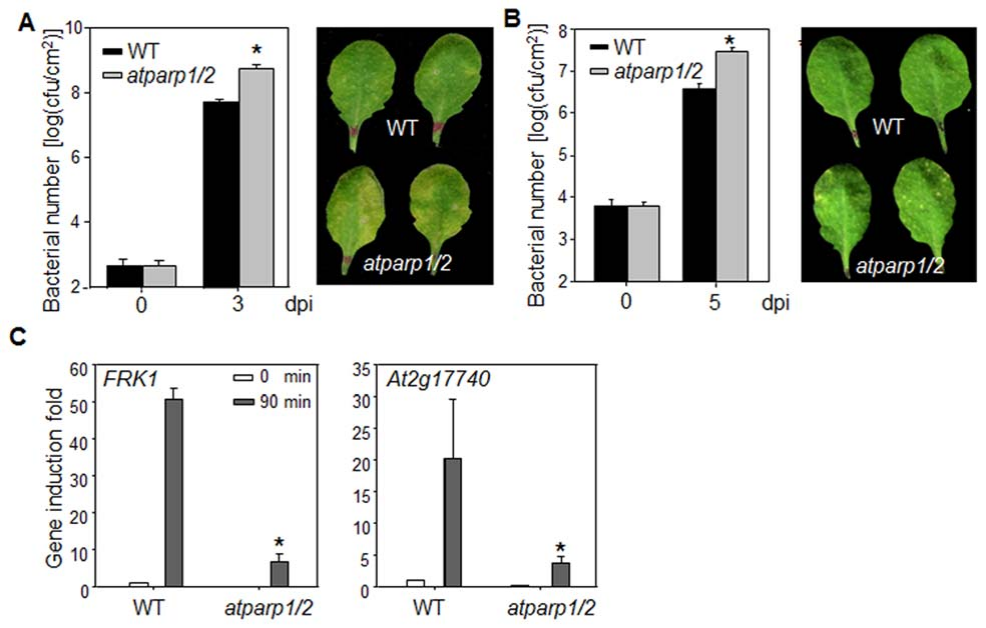
AtPARPs positively regulate *Arabidopsis* immunity. (**A**) The *atparp1/2* double mutant is more susceptible to *Psm* infection. WT (Col-0) and *atparp1/2* double mutant plants were hand-inoculated with *Psm* at OD_600_  =  5 × 10^−4^, and the bacterial counting was performed 0 and 3 days post-inoculation (dpi). The data are shown as mean ± se from three independent repeats with Student's *t*-test. * indicates p<0.05 when compared to WT (Left panel). The disease symptom is shown at 3 dpi (right panel). (**B**) The *atparp1/2* double mutant is more susceptible to *Pst* DC3000Δ*avrPtoavrPtoB* infection. WT and *atparp1/2* double mutant plants were hand-inoculated with *Pst* DC3000*ΔavrPtoavrPtoB* at OD_600_  =  5 × 10^-4^, and the bacterial counting was performed 0 and 5 dpi (left panel) and the disease symptom is shown at 5 dpi (right panel). (**C**) Reduced immune gene expression in *atparp1/2* mutant. Ten-day-old seedlings were treated with 100 nM flg22 for 90 min for qRT-PCR analysis. The data are shown as means ± se from three biological repeats. * indicates p<0.05 when compared to WT. The above experiments were repeated 4 times with similar results.

We tested the involvement of AtPARPs in plant innate immunity and immune gene activation. Because of the potential functional redundancy of AtPARP1 and AtPARP2 [30], [31], we performed disease assay and analyzed defense gene expression in *atparp1atparp2* (*atparp1/2*) double mutant. The *atparp1/2* mutant plants were more susceptible to virulent *P. syringae* pv. *maculicola* ES4326 (*Psm*) infection compared to WT plants as indicated by more than 10 fold increase of bacterial growth in the *atparp1/2* mutant (Fig. 6A). The disease symptom development was more pronounced in the *atparp1/2* mutant than WT plants (Fig. 6A). Similarly, the *atparp1/2* mutant plants showed the enhanced susceptibility with bacterial growth and symptom development to the infections by *Pst* DC3000 and a less virulent bacterium *Pst* DC3000*ΔavrPtoavrPtoB* (Fig. 6B & S7 Fig.). In addition, the *atparp1/2* mutant plants showed the reduced induction of MAMP marker genes, including *FRK1* and *At2g17740*, compared to WT plants at 90 min after flg22 treatment (Fig. 6C). Together, these data indicate that AtPARP1 and AtPARP2 are positive regulators in plant immunity and defense gene activation to bacterial infections.

## Discussion

Protein PARylation mediated by PARPs and PARGs is an important, but less understood posttranslational modification process implicated in the regulation of diverse cellular processes and physiological responses [26]. In this study, an unbiased genetic screen revealed that *Arabidopsis* AtPARG1 plays an important role in regulating immune gene expression upon pathogen infection. We established and performed extensive *in vitro* and *in vivo* biochemical assays of PARP and PARG enzymatic activities. We have shown for the first time that *Arabidopsis* AtPARP1 and AtPARP2 are able to transfer ADP-ribose moieties from NAD^+^ to itself and acceptor proteins *in vitro* and *in vivo*. Thus, they are *bona fide* poly(ADP-ribose) polymerases. Interestingly, in contrast to their mammalian counterparts, AtPARP2 is more enzymatically active than AtPARP1. Significantly, MAMP perception promotes substantial enhancement of AtPARP2 enzymatic activity *in vivo*, reconciling the biological importance of PARPs/PARGs in regulating immune gene expression. AtPARG1, but not AtPARG2, is able to remove PAR polymers from PARylated proteins *in vivo* and *in vitro* and it is a *bona fide* poly(ADP-ribose) glycohydrolase. The *Arabidopsis parg1* (*aggie2*) mutant plants exhibited elevated expression of several MAMP-induced genes and callose deposition. Conversely, the *Arabidopsis atparp1*/*2* mutant showed reduced expression of MAMP-induced genes and enhanced susceptibility to virulent *Pseudomonas* infections. Thus, the data suggest that protein PARylation positively regulates certain aspects of plant immune responses. Notably, the viability and normal growth of *Arabidopsis parp* and *parg* null mutants represent a unique opportunity to study protein PARylation regulatory mechanisms in diverse biological processes at the whole organismal level.

Our results lend support to a previous study that treatment of pharmacological inhibitor of PARPs, 3-AB, disrupted elf18- and/or flg22-induced callose and lignin deposition, pigment accumulation and phenylalanine ammonia lyase activity [37]. However, the flg22-induced defense genes (*FRK1* and *WRKY29*) were not affected by 3-AB treatment [37]. Our study with *Arabidopsis parg* and *parp* genetic mutants revealed a previously unrecognized function of protein PARylation in regulating immune gene expression upon pathogen infection. This is consistent with the general role of human PARPs and PARG in transcriptional regulation and chromatin modification [43], [45] and further substantiates the hypothesis that plant PARPs could ameliorate the cellular stresses caused by antimicrobial defenses (e.g. the effects of elevated ROS levels) [23]. Interestingly, ADP-ribosylation has also been exploited by pathogens as a means to quell plant immunity. Two *Pseudomonas syringae* effectors, HopU1 and HopF2, mono-ADP-ribosylate RNA-binding protein GRP7 and MAPK kinase MKK5 respectively, and interfere with their activities in plant defense transcription regulation and signaling [46], [47].

Unlike mammals and most other animals that encode a single *PARG* gene, the *Arabidopsis* genome encodes two adjacent *PARG* genes, *AtPARG1* and *AtPARG2*, as well as a pseudogene *At2g31860*. Surprisingly, only AtPARG1, but not AtPARG2, possesses detectable poly(ADP-ribose) glycohydrolase activity *in vitro* and *in vivo* with our extensive biochemical assays. Sequence analysis identified a polymorphism in the conserved PARG signature motif “GGG-X_7_-QEE”, where the third G is replaced with an L in AtPARG2. The PARG signature motif is absolutely required for its enzymatic activity as mutations at this motif in AtPARG1 completely abolished its activity. However, creation of the conserved signature motif in AtPARG2 was unable to gain its PARG activity suggesting that other polymorphisms in AtPARG2 are also responsible for its lack of enzymatic activity. Consistent with our biochemical assays, the PAR polymer concentration was much higher in *atparg1* mutant than that in WT plants. A similar conclusion was reached on *tej* mutant, which carries a G262E mutation in the invariable signature motif of AtPARG1 [35]. The *atparg1*, but not *atparg2* mutant, affected elf18-induced seedling growth inhibition and pigment formation, and sensitivity to DNA-damaging agent [37]. Interestingly, *AtPARG2* is substantially induced in multiple plant-pathogen interactions [36] and it is required for plant resistance to *B. cinerea* infections [37]. Thus, despite of lacking detectable enzymatic activity, AtPARG2 may still play certain role in plant immunity. It is possible that AtPARG2 may regulate AtPARG1 activity. It is also possible that AtPARG2 has evolved novel functions in plant immune responses. Several other plant species, including rice, poplar, tomato and maize, are also predicted to encode multiple PARGs [23] (S8 Fig.). Unlike *Arabidopsis* PARGs, different PARG members in other species have invariant signature motif. For example, all three PARGs in poplar contain GGG-X_7_-QEE signature motif (S8 Fig.). However, a few other species such as *Eutrema salsugineum*, *Capsella rubella*, *Phaseolus vulgaris*, *Oikopleura dioica*, and *Xenopus laevis*, contain PARGs with an AtPARG2-like signature GGL-X_7_-QEE. It remains unknown how many PARGs are enzymatic active in the species with multiple PARGs.

Although there are 17 PARPs in mammals, the *parp-1parp-2* double mutant mice are not viable and die at the onset of gastrulation, suggesting the essential role of protein PARylation during early embryogenesis [48]. The lethality of *parp-1parp-2* double mutant mice might be due to genomic instability. However, *Arabidopsis atparp1/2* double mutant is largely morphologically similar with WT plants and does not display any obvious growth defects. Although *Arabidopsis atparp1/2* double mutant was hypersensitive to genotoxic stress, they did not have significant changes in telomere length nor end-to-end chromosome fusions [30]. Albeit mainly expressed in developing seeds, AtPARP3 may have redundant functions with AtPARP1 and AtPARP2 in maintaining genome stability. It remains interesting whether *atparp1/2/3* triple mutant will exert abnormal plant growth and development. Consistent with the essential function of PARylation during embryogenesis, PARG-deficient mice and *Drosophila* are embryonic lethal which is probably due to the accumulation of PAR polymers and uncontrolled PAR-dependent signaling [49], [50]. The normal plant growth phenotype of *atparg1* mutant might be due to the redundant function of AtPARG2. However, our extensive biochemical analysis indicates that AtPARG1, but not AtPARG2, accounts for most of PARG enzymatic activity. As *AtPARG1* and *AtPARG2* reside next to each other on the same chromosome, it is challenging to generate the double mutant. It remains possible that other PAR-degrading enzymes with distinct sequences exist in *Arabidopsis*. In vertebrate, ADP-ribosyl hydrolase 3 (ARH3), a structurally distinct enzyme from PARG, could also degrade PAR polymers associated with the mitochondrial matrix [26].

We observed that AtPARP2 activity was rapidly and substantially stimulated by flg22 treatment. In line with this observation, it has been shown that bacterial infections induced the increase of PAR polymers in *Arabidopsis* [37]. It is well established that damaged DNA stimulates PARP activity. Recent studies have shown that pathogen treatments induce DNA damage [51], [52], which could potentially serve as a trigger to activate PARP. Treatments with virulent or avirulent *Pst* strains for hours could induce DNA damage in *Arabidopsis* as detected by abundance of histone γ-H2AX, a sensitive indicator of DNA double-strand breaks or by DNA comet assays [51]. Prolonged pathogen treatment is often accompanied with the elevated accumulation of plant defense hormone salicylic acid (SA). It has also been shown that SA can also trigger DNA damage in the absence of a genotoxic agent [53]. However, treatments of flg22 or elf18 did not induce detectable DNA damage [51]. In addition, flg22-mediated stimulation of AtPARP2 activity occurs rather rapidly and within 30 min after treatment. Apparently, flg22 signaling could directly activate AtPARP2. It is well known that human HsPARP-1 is regulated by different posttranslational modification processes, such as phosphorylation, ubiquitination, SUMOylation and cleavage [22]. HsPARP-1 could be activated by phosphorylated MAPK ERK2 in a broken DNA-independent manner, thereby enhancing ERK-induced Elk1 phosphorylation, core histone acetylation, and transcription of the Elk1-target genes [54]. MAPK cascade plays a central role functioning downstream of multiple MAMP receptors. It will be interesting to test whether flg22-activated MAPKs directly modulate PARP and/or PARG activities.

Our genetic and biochemical analyses revealed that PARP/PARG-mediated PAR dynamics regulates immune gene expression in *Arabidopsis*. Mammalian PARPs/PARG regulate gene expression through a variety of mechanisms including modulating chromatin, functioning as transcriptional co-regulators and mediating DNA methylation [55]. PARylation of histone lysine demethylase KDM5B maintains histone H3 lysine 4 trimethyl (H3K4me3), a histone mark associated with active promoters, by inhibiting KDM5B demethylase activity and interactions with chromatin. In addition, HsPARP-1 is able to promote exclusion of H1 and opening of promoter chromatin, which collectively lead to a permissive chromatin environment that allows loading of the RNAPII machinery [45]. HsPARG is also able to promote the formation of a chromatin environment suitable for retinoic acid receptor (RAR)-mediated transcription by removing PAR polymer from PARylated H3K9 demethylase KDM4D/JMJD2D thereby activating KDM4D/JMJD2D to inhibit H3K9me2, a histone mark associated with transcriptional repression [43]. *Arabidopsis* PARPs and PARGs are localized in the nucleus, and AtPARP2 could PARylate Histone H1. It is plausible to speculate that similar modes of action of protein PARylation-mediated transcriptional regulation exist in plants. Future identification of PARP/PARG targets (promoters and proteins) and PAR-associated proteins, especially during plant immune responses, will elucidate how protein PARylation modulates plant immune gene expression.

## Materials and Methods

### Plant and pathogen materials and growth conditions

*Arabidopsis* accession Col-0, *pFRK1::LUC* transgenic plants, *aggie2* mutant, *atparg1-1* (SALK_147805), *atparg1-2* (SALK_16088), *atparg2* (GABI-Kat 072B04), *atparp1/atparp2* (GABI-Kat 692A05/SALK_640400), *pPARG1::PARG1-FLAG* transgenic plants were grown in soil (Metro Mix 366) at 23°C, 60% humidity and 75 µE m^−2^s^−1^ light with a 12-hr light/12-hr dark photoperiod. Four-week-old plants were used for protoplast isolation and transient expression assays according to the standard procedure [56]. Seedlings were germinated on ½ Murashige and Skoog (MS) plate containing 1% sucrose, 0.8% Agar and grown at 23°C and 75 µE m^-2^s^−1^ light with a 12-hr light/12-hr dark photoperiod for 12 days, transferred to a 6-well tissue culture plate with 2 ml H_2_O for overnight, and then treated with 100 nM flg22 or H_2_O for indicated time.

*Pseudomonas syringae* pv. *tomato* (*Pst*) DC3000, *hrcC, ΔavrPtoavrPtoB, P. syringae* pv. *maculicola* ES4326 (*Psm*), or *P. syringae* pv. *phaseolicola* NPS3121 strains were cultured overnight at 28°C in the KB medium with 50 µg/ml rifampicin or streptomycin. Bacteria were harvested by centrifugation, washed, and adjusted to the desired density with 10 mM MgCl_2_. Leaves of 4-week-old plants were hand-infiltrated with bacterial suspension using a 1-ml needleless syringe and collected at the indicated time for luciferase activity or bacterial growth assays. To measure bacterial growth, two leaf discs were ground in 100 µl H_2_O and serial dilutions were plated on TSA medium (1% Bacto tryptone, 1% sucrose, 0.1% glutamic acid, 1.5% agar) with appropriate antibiotics. Bacterial colony forming units (cfu) were counted 2 days after incubation at 28°C. Each data point is shown as triplicates. *Botrytis cinerea* strain BO5 was cultured on Potato Dextrose Agar (Difco) and incubated at room temperature. Conidia were re-suspended in distilled water and spore concentration was adjusted to 2.5 × 10^5^ spores/ml. Gelatin (0.5%) was added to conidial suspension before inoculation. Leaves of six-week-old plants were drop-inoculated with *B. cinerea* at the concentration of 2.5 × 10^5^ spores/ml. Lesion size was measured 2 days post-inoculation.

### Mutant screening, map-based cloning and next generation sequencing

The *pFRK1::LUC* construct in a binary vector was transformed into *Arabidopsis* Col-0 plants. The homozygous transgenic plants with flg22-inducible *pFRK1::LUC* were selected for mutagenesis. The seeds were mutagenized with 0.4% ethane methyl sulfonate (EMS). Approximately 6,000 M2 seedlings were screened for their responsiveness to flg22 treatment. The seedlings were germinated in liquid ½ MS medium for 14 days, and then transferred to water for overnight and treated with 10 nM flg22. After 12 hr flg22 treatment, the individual seedlings were transferred to a 96-well plate, sprayed with 0.2 mM luciferin and kept in dark for 20 min. The bioluminescence from induced *pFRK1::LUC* expression was recorded by a luminometer (Perkin Elmer, 2030 Multilabel Reader, Victor X3). The candidate mutants with altered flg22 responsiveness were recovered on ½ MS plate for 10 days, and then transferred to soil for seeds.

The *aggie2* mutant was crossed with *Arabidopsis Ler* accession, and an F2 population was used for map-based cloning. Mapping with 270 F2 plants with *aggie2* mutant phenotype placed the causal mutation in an 88 kb region between marker F20F17 and F22D22 on chromosome 2. The *aggie2* genomic DNA was sequenced with the 100 nt paired-end sequencing on an Illumina HiSeq 2000 platform at Texas AgriLife Genomics and Bioinformatics Service (TAGS) (College Station, TX, USA). Ten-fold genome coverage was obtained with 11M reads. The Illumina reads were analyzed using CLC Genomics Workbench 6.0.1 software. By mapping to Col-0 genomic sequence (TAIR10 release), SNPs were identified as candidates of *aggie2* mutation. In the aforementioned 88 kb region, a G to A mutation at the position of 1348 nt of At2g31870 was identified with 100% frequency. The mutation was confirmed by Sanger sequencing of *aggie2* genomic DNA.

### Plasmid constructs for protoplasts and transgenic plants

The *AtPARP1, AtPARP2, AtPARG1, AtPARG2* and Histone *H1.1* (*At1g06760*) genes were amplified from *Arabidopsis* Col-0 cDNA and cloned into a plant transient expression vector (pHBT vector) with an HA, FLAG or GFP epitope tag at the C-terminus via restriction sites NcoI or BamHI and StuI respectively. The oligos used to amplify aforementioned cDNAs are listed in S1 Table. The target genes were confirmed by Sanger sequencing. The cloned genes in plant expression vector were then sub-cloned into protein fusion vectors, pGEX-4T (Pharmacia, USA), pMAL-c (NEB, USA) or pET28a (EMD Millipore, USA), for protein expression in bacteria. For Histone H1.3 (At2g18050), we ordered cDNA from ABRC (G13366) and cloned it into a modified pMAL-c via SfiI site. Point mutations were introduced by site-directed mutagenesis PCR. The *AtPARG1* promoter (1163 bp upstream of start codon ATG) was amplified from the genomic DNA of Col-0 and digested with KpnI and NcoI. The AtPARG1-FLAG-NOS terminator fragment was released from pHBT-AtPARG1-FLAG via NcoI and EcoRI digestion. The two fragments were ligated and sub-cloned into a binary vector, pCAMBIA2300 via KpnI and EcoRI sites to yield expression construct (*pAtPARG1::AtPARG1-FLAG*). The resulting binary vector was transformed into *aggie2* via *Agrobacterium*-mediated transformation.

The primers for cloning and point mutations were listed in the S1 Table.

### *In vitro* and *in vivo* PARP and PARG assays

Expression and purification of GST, His6 and MBP fusion proteins were performed according to the manufacturer's manuals. For *in vitro* auto-PARylation reaction, 1.2 µg of MBP-AtPARP2 or MBP-AtPARP1 proteins were incubated in a 20 µl reaction with 1 × PAR reaction buffer (50 mM Tris-HCl, pH8.0, 50 mM NaCl) with 0.2 mM NAD^+^, and 1 × activated DNA (Trevigen, USA). To inhibit PAR reaction, 2.5 mM PARP inhibitor, 3-Aminobenzamide (3-AB, Sigma, USA), was added to the reaction. The reactions were kept at room temperature for 30 min and stopped by adding SDS loading buffer. To detect PARG activity, about 1.0 µg of purified GST, GST-AtPARG1 or GST-AtPARG2 proteins together with 2.5 mM 3-AB were added to auto-PARylated AtPARP2 proteins derived from the above PAR reactions and incubated at room temperature for another 30 min. PARylated proteins were separated in 7.5% SDS-PAGE and detected with an α-PAR polyclonal antibody (Trevigen, USA). For Biotin NAD^+^ PAR assay, 25 µM Biotin-NAD^+^ (Trevigen, USA) was added to replace NAD^+^ in the reaction described above. The PAR polymer formation was detected by Streptavidin-HRP (Pierce, USA). For *in vitro* ^32^P-NAD^+^-mediated PAR assays, 1.0 µg of MBP-AtPARP2 or MBP-AtPARP1 proteins were incubated in a 20 µl reaction in the buffer containing 50 mM Tris-HCl, pH8.0, 4 mM MgCl_2_, 300 mM NaCl, 1 mM DTT, 0.1 µg/ml BSA, 1 × activated DNA, 1 µCi ^32^P-NAD^+^ (Perkin Elmer, USA) and 100 nM cold NAD^+^ for 30 min at room temperature. For Histone PARylation assays, 2.0 µg of MBP-H1.1 or MBP-H1.3 proteins were added in the above reactions. The radiolabeled proteins were separated in SDS-PAGE and visualized by autoradiography.

For *in vivo* PAR assays, 500 µl *Arabidopsis* protoplasts at the concentration of 2 × 10^5^/ml were transfected with 100 µg of plasmid DNA of pHBT-AtPARP2-HA. After 12 hr incubation, the protoplasts were treated with 100 nM flg22 for 30 min and fed with 1 µCi ^32^P-NAD^+^ for 1 hr. The protoplasts were then lysed in IP buffer (50 mM Tris-HCl, pH7.5, 150 mM NaCl, 5 mM EDTA, 1% Triton, 1 × protease inhibitor, 1 mM DTT, 2 mM NaF and 2 mM Na_3_VO_4_) and the AtPARP2-HA proteins were immunoprecipitated with α-HA antibody (Roche, USA) and protein-G-agarose (Roche, USA) in a shaker for 3 hr at 4°C. *In vivo* PARylated proteins enriched on the beads were then separated in 10% SDS-PAGE and visualized by autoradiography. For *in vivo* PARG assay, AtPARG1-HA or AtPARG2-HA plasmid DNA was co-transfected with AtPARP2-FLAG plasmid DNA into protoplasts, and expressed for 12 hr. The protoplasts were fed with ^32^P-NAD^+^ and subjected to immunoprecipitation as described above. The AtPARP2-FLAG proteins were immunoprecipitated with α-FLAG agarose gel (Sigma, USA), separated in 10% SDS-PAGE and visualized by autoradiography. The expression of AtPARPs and AtPARGs was detected with Western blot (WB) using the corresponding antibodies.

### Detection of PAR polymers from protein extract of nuclei

The 12-day old seedlings grown on ½ MS plates were harvested and ground into fine powder in liquid nitrogen. Isolation of nuclei with Honda buffer was performed according to published procedure [57]. Nuclear proteins were released in 1xPBS buffer with 1% SDS and spotted on nitrocellulose membrane. The protein loaded on the membrane was normalized by using α-Histone H3 antibody (Abcam, USA), and the PAR polymers were detected by α-PAR antibody. The relative PAR level was determined by calculating the ratio of PAR signal to Histone H3 signal after quantification of hybridization intensity with ImageJ software.

### RNA isolation and RT-PCR

For RNA isolation, 12-day-old seedlings grown on ½ MS plate were transferred to 2 ml H_2_O in a 6-well plate to recover for 1 day, and then treated with 100 nm flg22 for 30 or 90 min. RNA was extracted using TRIzol reagent (Life Technologies, USA) and quantified with NanoDrop. The RNA was treated with RQ1 RNase-free DNase I (Promega, USA) for 30 min at 37°C, and then reverse transcribed with M-MuLV Reverse Transcriptase (NEB, USA). Real-time RT-PCR was carried out using iTaq Universal SYBR Green Supermix (Bio-Rad, USA) on 7900HT Fast Real-Time PCR System (Applied Biosystems, USA). The primers used to detect specific transcript by real-time RT-PCR are listed in S2 Table.

### Callose deposition

Leaves of six-week-old plants grown in soil were hand-inoculated with 0.5 µM flg22 or H_2_O for 12 hr. The leaves were then transferred into FAA solution (10% formaldehyde, 5% acetic acid and 50% ethanol) for 12 hr, de-stained in 95% ethanol for 6 hr, washed twice with ddH_2_O, and incubated in 0.01% aniline blue solution (150 mM KH_2_PO4, pH 9.5) for 1 hr. The callose deposits were visualized with a fluorescence microscope. Callose deposits were counted using ImageJ 1.43U software (http://rsb.info.nih.gov/ij/).

### Lignin deposition

Leaves of six-week-old plants grown in soil were surface-sterilized by 70% ethanol, rinsed with H_2_O and incubated with 100 nM flg22 or H_2_O for 12 hr. The leaves were then de-stained in 95% ethanol with 2% chloroform for 12 hr and 95% ethanol for 6 hr, washed twice with 95% ethanol, and incubated in 2% phloroglucinol solution (20% ethanol, 20% HCl) for 5 min. The images were scanned by HP officejet Pro 8600 Premium.

### MAPK assay

Ten-day-old seedlings germinated on ½MS plate were transferred to 2ml H_2_O in a 6-well plate to recover for 1 day, and then treated with 100 nM flg22 for 5, 15 or 45 min. The seedlings were grinded in IP buffer. The cleared lysate was mixed with SDS sample buffer and loaded onto 12.5% SDS-PAGE. Activated MAPKs were detected with α-pErk1/2 antibody (Cell Signaling, USA).

### ROS analyses

ROS burst was determined by a luminol-based assay. At least 10 leaves of four-week-old *Arabidopsis* plants for each genotype were excised into leaf discs of 0.25 cm^2^, followed by an overnight incubation in 96-well plate with 100 µl of H_2_O to eliminate the wounding effect. H_2_O was replaced by 100 µl of reaction solution containing 50 µM luminol and 10 µg/ml horseradish peroxidase (Sigma, USA) supplemented with or without 100 nM flg22. The measurement was conducted immediately after adding the solution with a luminometer (Perkin Elmer, 2030 Multilabel Reader, Victor X3), with a 1.5 min interval reading time for a period of 30 min. The measurement values for ROS production from 20 leaf discs per treatment were indicated as means of RLU (Relative Light Units).

### GFP localization assay

*Arabidopsis* protoplasts were transfected with various GFP-tagged pHBT constructs as indicated in the figures. Fluorescence signals in the protoplasts were visualized under a confocal microscope 12 hr after transfection. To construct *35S::AtPARP2-GFP* binary plasmid for *Agrobacterium*-mediated transient assay, the NcoI-PstI fragment containing *AtPARP2-GFP* was released from *pHBT-35S::AtPARP2-GFP* and ligated into pCB302 binary vector. For tobacco transient expression, *Agrobacterium tumefaciens* strain GV3101 containing *pCB302-35S::AtPARP2-GFP* was cultured at 28°C for 18 hr. Bacteria were harvested by centrifugation at a speed of 3500 rpm and re-suspended with infiltration buffer (10 mM MES pH = 5.7, 10 mM MgCl_2_, 200 µM acetosyringone). Cell solution at OD600 = 0.75 was used to infiltrate 3-week-old *Nicotiana benthamiana* leaves. Fluorescence signals were detected 2 days post-infiltration. Fluorescence images were taken with Nikon-A1 confocal laser microscope systems and images were processed using NIS-Elements Microscope Imaging Software. The excitation lines for imaging GFP, RFP and chloroplast were 488, 561 and 640 nm, respectively.

## Supporting Information

S1 Fig PARP- and PARG-mediated posttranslational PARylation in cellular stress responses. Extrinsic and intrinsic stress signals activate PARP which transfers ADP-ribose moiety from NAD+ to acceptor proteins resulting in the formation of linear or branched poly(ADP-ribose) (PAR) polymers. PARG could also be activated by different stresses and remove PAR polymers from acceptor proteins. Nucleoside diphosphate linked to some moiety-X (NUDX) then cleaves free ADP-ribose into AMP (adenosine monophosphate) and ribose-5-phosphate.
(TIF)

S2 Fig Domain organization of PARPs and PARGs. **(A)** Domain organization of human HsPARPs and *Arabidopsis* AtPARPs; ZFI: PARP-like zinc-finger1 domain; ZFII: PARP-like zinc-finger2 domain; ZF(PADR): zinc-binding domain 3; BRCT: BRCA1 carboxy-terminal domain for protein–protein and protein–DNA break binding domain; WGR: Trp-Gly-Arg in single letter code for putative PARP nucleic acid binding domain; PRD: PARP regulatory domain; PARP: PARP catalytic domain; SAP: SAF-A/B, Acinus and PIAS motif for putative DNA/RNA binding domain; **(B)** Domain structure of human HsPARG, Rat RnPARG and *Arabidopsis* AtPARGs. A-domain: N-terminal regulatory and targeting domain; MTS: mitochondrial targeting sequence; Macrodomain fold: core catalytic domain. The number under each domain indicates the position of amino acid in the protein.
(TIF)

S3 Fig Disease assays in *aggie2*. **(A)** The *aggie2* mutant response to *Pst* DC3000 infection. WT and *aggie2* mutant plants were hand-inoculated with *Pst* DC3000 at OD_600_  =  5 × 10^-4^, and the bacterial counting was performed 3 days post-inoculation (dpi). The data are shown as mean ± se from three independent repeats. We performed 7 times of disease assays, and observed that *aggie2* was more resistant than WT plants for 4 times, and there is no difference between *aggie2* and WT for other 3 times. The representative bacterial counting with difference (left) or without difference (right) is shown. **(B)** The *aggie2* mutant is more susceptible to *B. cinerea* infection. Leaves of six-week-old plants were drop-inoculated with *B. cinerea* at the concentration of 2.5 × 10^5^ spores/ml. Lesion size was measured 2 days post-inoculation. The data are shown as mean ± se from 20 infected leaves.
(TIF)

S4 Fig Expression pattern of *AtPARG1, AtPARG2, AtPARP1, AtPARP2* and *AtPARP3*. **(A)** Response of *AtPARG1* and *AtPARG2* transcript level to flg22 treatment. The 12-day-old seedlings were treated with 100nM flg22 for qRT-PCR analysis. **(B)** The transcript levels of *AtPARP1*, *AtPARP2* and *AtPARP3* in 6 primary organs (Rt, root; St, Stem; Lf, leaf; Inf, inflorescence; Sq, silique; Sd, seeds) detected by qRT-PCR. *AtPARP1* and *AtPARP2* are expressed in all 6 organs. However, *AtPARP3* is predominantly expressed in seeds but not in other organs **(C)** In silicon analysis of *AtPARP1, 2, 3* and *AtPARG1*. The figures were obtained from *Arabidopsis* eFP Browser (http://bbc.botany.utoronto.ca/efp/cgi-bin/efpWeb.cgi) with indicated AGI numbers. Winter *et al*., 2007. PLoS One 2(8): e718.
(TIF)

S5 Fig *In vitro* and *in vivo* activity of PARPs and PARGs. **(A)** The *in vitro* PARP activity of human HsPARP-1 and *Arabidopsis* AtPARP2 detected by an α-PAR Western blot. **(B)** *In vitro* enzymatic activity of His6-tagged AtPARG1 and AtPARG2. His6-AtPARG1, but not His6-AtPARG2, hydrolyzed PAR polymers from self-modified MBP-AtPARP2 shown as the disappearance of smear detected by α-PAR antibody. **(C)** GST-AtPARG1 hydrolyzes PAR polymers from self-modified AtPARP2 and HsPARP-1, and aggie2 mutation (G450R) blocks its activity. **(D)** HsPARGG867R, the corresponding mutation in aggie2, abolishes its PARG activity towards self-modified AtPARP2. **(E)**. *In vivo* PAR level in Col-0, *atparg1, atparg2* and *atparp1/2*. Nuclear protein extracts were isolated, dotted onto nitrocellulose membrane, probed with α-PAR antibody (left), and quantified with ImageJ software (right). Amount of nuclear proteins was normalized to the signal of α-Histone H3 antibody WB. The *atparg1* mutant accumulates higher PAR polymers than Col-0; however, PAR polymer level in *atparg2* is comparable with that in Col-0.
(TIF)

S6 Fig Comparison of AtPARG1 and AtPARG2 amino acid sequences. The alignment was generated with "Multiple sequence alignment with hierarchical clustering" F. CORPET, 1988, Nucl. Acids Res., 16 (22), 10881-10890. (http://multalin.toulouse.inra.fr/multalin/)
(TIF)

S7 Fig The *atparp1/2* mutant is more susceptible to *Pst* DC3000 infection. WT and *atparp1/2* double mutant plants were hand-inoculated with *Pst* DC3000 at OD_600_  =  5 × 10^-4^, and the bacterial counting was performed 3 days post-inoculation (dpi). The data are shown as mean ± se from three independent repeats with Student's *t*-test. * indicates p<0.05 when compared to WT (Left panel). The disease symptom is shown at 3 dpi (right panel).
(TIF)

S8 Fig Sequence alignment of PARG signature motif among PARGs from different species. thale cress (*Arabidopsis thaliana*, At), lyrate rockcress (*Arabidopsis lyrata*, Al), poplar (*Populus trichocarpa*, Pt), potato (*Solanum tuberosum*, St), tomato (*Solanum lycopersicum*, Sl), maize(Zea mays, Zm), sorghum (*Sorghum bicolor*, Sb), rice (*Oryza sativa*, Os), moss(*Physcomitrella patens*, Pp), rat (*Rattus norvegicus*, Rn), mouse(*Mus musculus*, Mm), human (*Homo sapiens*, Hs), fruit fly (*Drosophila melanogaster*, Dm). The PARG signature motif is labeled in red.
(TIF)

S1 Table Cloning and point mutation primers
(DOCX)

S2 Table qRT-PCR primers
(DOCX)

## References

[1] Boller T, Felix G (2009) A renaissance of elicitors: perception of microbe-associated molecular patterns and danger signals by pattern-recognition receptors. Annu Rev Plant Biol 60: 379–406. 19400727 10.1146/annurev.arplant.57.032905.105346

[2] Dodds PN, Rathjen JP (2010) Plant immunity: towards an integrated view of plant-pathogen interactions. Nat Rev Genet 11: 539–548. 20585331 10.1038/nrg2812

[3] Macho AP, Zipfel C (2014) Plant PRRs and the activation of innate immune signaling. Mol Cell 54: 263–272. 24766890 10.1016/j.molcel.2014.03.028

[4] Chinchilla D, Bauer Z, Regenass M, Boller T, Felix G (2006) The Arabidopsis receptor kinase FLS2 binds flg22 and determines the specificity of flagellin perception. Plant Cell 18: 465–476. 16377758 10.1105/tpc.105.036574 PMC1356552

[5] Gomez-Gomez L, Boller T (2000) FLS2: an LRR receptor-like kinase involved in the perception of the bacterial elicitor flagellin in Arabidopsis. Mol Cell 5: 1003–1011. 10911994 10.1016/s1097-2765(00)80265-8

[6] Heese A, Hann DR, Gimenez-Ibanez S, Jones AM, He K, et al (2007) The receptor-like kinase SERK3/BAK1 is a central regulator of innate immunity in plants. Proc Natl Acad Sci U S A 104: 12217–12222. 17626179 10.1073/pnas.0705306104 PMC1924592

[7] Chinchilla D, Zipfel C, Robatzek S, Kemmerling B, Nurnberger T, et al (2007) A flagellin-induced complex of the receptor FLS2 and BAK1 initiates plant defence. Nature 448: 497–500. 17625569 10.1038/nature05999

[8] Schulze B, Mentzel T, Jehle AK, Mueller K, Beeler S, et al (2010) Rapid heteromerization and phosphorylation of ligand-activated plant transmembrane receptors and their associated kinase BAK1. J Biol Chem 285: 9444–9451. 20103591 10.1074/jbc.M109.096842 PMC2843194

[9] Sun Y, Li L, Macho AP, Han Z, Hu Z, et al (2013) Structural basis for flg22-induced activation of the Arabidopsis FLS2-BAK1 immune complex. Science 342: 624–628. 24114786 10.1126/science.1243825

[10] Lu D, Wu S, Gao X, Zhang Y, Shan L, et al (2010) A receptor-like cytoplasmic kinase, BIK1, associates with a flagellin receptor complex to initiate plant innate immunity. Proc Natl Acad Sci U S A 107: 496–501. 20018686 10.1073/pnas.0909705107 PMC2806711

[11] Zhang J, Li W, Xiang T, Liu Z, Laluk K, et al (2010) Receptor-like cytoplasmic kinases integrate signaling from multiple plant immune receptors and are targeted by a Pseudomonas syringae effector. Cell Host Microbe 7: 290–301. 20413097 10.1016/j.chom.2010.03.007

[12] Lin W, Li B, Lu D, Chen S, Zhu N, et al (2014) Tyrosine phosphorylation of protein kinase complex BAK1/BIK1 mediates Arabidopsis innate immunity. Proc Natl Acad Sci U S A 111: 3632–3637. 24532660 10.1073/pnas.1318817111 PMC3948311

[13] Xu JH, Wei XC, Yan LM, Liu D, Ma YY, et al (2013) Identification and functional analysis of phosphorylation residues of the Arabidopsis BOTRYTIS-INDUCED KINASE1. Protein & Cell 4: 771–781. 24104392 10.1007/s13238-013-3053-6 PMC4875430

[14] Roux M, Schwessinger B, Albrecht C, Chinchilla D, Jones A, et al (2011) The Arabidopsis leucine-rich repeat receptor-like kinases BAK1/SERK3 and BKK1/SERK4 are required for innate immunity to hemibiotrophic and biotrophic pathogens. Plant Cell 23: 2440–2455. 21693696 10.1105/tpc.111.084301 PMC3160018

[15] Postel S, Kufner I, Beuter C, Mazzotta S, Schwedt A, et al (2010) The multifunctional leucine-rich repeat receptor kinase BAK1 is implicated in Arabidopsis development and immunity. Eur J Cell Biol 89: 169–174. 20018402 10.1016/j.ejcb.2009.11.001

[16] Liu Z, Wu Y, Yang F, Zhang Y, Chen S, et al (2013) BIK1 interacts with PEPRs to mediate ethylene-induced immunity. Proc Natl Acad Sci U S A 110: 6205–6210. 23431184 10.1073/pnas.1215543110 PMC3625333

[17] Li J, Wen J, Lease KA, Doke JT, Tax FE, et al (2002) BAK1, an Arabidopsis LRR receptor-like protein kinase, interacts with BRI1 and modulates brassinosteroid signaling. Cell 110: 213–222. 12150929 10.1016/s0092-8674(02)00812-7

[18] Nam KH, Li J (2002) BRI1/BAK1, a receptor kinase pair mediating brassinosteroid signaling. Cell 110: 203–212. 12150928 10.1016/s0092-8674(02)00814-0

[19] Lin W, Lu D, Gao X, Jiang S, Ma X, et al (2013) Inverse modulation of plant immune and brassinosteroid signaling pathways by the receptor-like cytoplasmic kinase BIK1. Proc Natl Acad Sci U S A 110: 12114–12119. 23818580 10.1073/pnas.1302154110 PMC3718091

[20] Kadota Y, Sklenar J, Derbyshire P, Stransfeld L, Asai S, et al.. (2014) Direct Regulation of the NADPH Oxidase RBOHD by the PRR-Associated Kinase BIK1 during Plant Immunity. Mol Cell. 10.1016/j.molcel.2014.02.021 24630626

[21] Li L, Li M, Yu L, Zhou Z, Liang X, et al (2014) The FLS2-Associated Kinase BIK1 Directly Phosphorylates the NADPH Oxidase RbohD to Control Plant Immunity. Cell Host Microbe 15: 329–338. 24629339 10.1016/j.chom.2014.02.009

[22] Luo X, Kraus WL (2012) On PAR with PARP: cellular stress signaling through poly(ADP-ribose) and PARP-1. Genes Dev 26: 417–432. 22391446 10.1101/gad.183509.111 PMC3305980

[23] Briggs AG, Bent AF (2011) Poly(ADP-ribosyl)ation in plants. Trends Plant Sci 16: 372–380. 21482174 10.1016/j.tplants.2011.03.008

[24] Kalisch T, Ame JC, Dantzer F, Schreiber V (2012) New readers and interpretations of poly(ADP-ribosyl)ation. Trends Biochem Sci 37: 381–390. 22766145 10.1016/j.tibs.2012.06.001 PMC7127722

[25] Kraus WL, Lis JT (2003) PARP goes transcription. Cell 113: 677–683. 12809599 10.1016/s0092-8674(03)00433-1

[26] Gibson BA, Kraus WL (2012) New insights into the molecular and cellular functions of poly(ADP-ribose) and PARPs. Nature Reviews Molecular Cell Biology 13: 411–424. 22713970 10.1038/nrm3376

[27] Kim IK, Kiefer JR, Ho CM, Stegeman RA, Classen S, et al (2012) Structure of mammalian poly(ADP-ribose) glycohydrolase reveals a flexible tyrosine clasp as a substrate-binding element. Nat Struct Mol Biol 19: 653–656. 22609859 10.1038/nsmb.2305 PMC3381899

[28] Slade D, Dunstan MS, Barkauskaite E, Weston R, Lafite P, et al (2011) The structure and catalytic mechanism of a poly(ADP-ribose) glycohydrolase. Nature 477: 616–620. 21892188 10.1038/nature10404 PMC3184140

[29] Lamb RS, Citarelli M, Teotia S (2012) Functions of the poly(ADP-ribose) polymerase superfamily in plants. Cell Mol Life Sci 69: 175–189. 21861184 10.1007/s00018-011-0793-4 PMC11114847

[30] Boltz KA, Jasti M, Townley JM, Shippen DE (2014) Analysis of poly(ADP-Ribose) polymerases in Arabidopsis telomere biology. PLoS One 9: e88872. 24551184 10.1371/journal.pone.0088872 PMC3923816

[31] Jia Q, den Dulk-Ras A, Shen H, Hooykaas PJ, de Pater S (2013) Poly(ADP-ribose)polymerases are involved in microhomology mediated back-up non-homologous end joining in Arabidopsis thaliana. Plant Mol Biol 82: 339–351. 23625359 10.1007/s11103-013-0065-9

[32] Schulz P, Jansseune K, Degenkolbe T, Meret M, Claeys H, et al (2014) Poly(ADP-ribose)polymerase activity controls plant growth by promoting leaf cell number. PLoS One 9: e90322. 24587323 10.1371/journal.pone.0090322 PMC3938684

[33] De Block M, Verduyn C, De Brouwer D, Cornelissen M (2005) Poly(ADP-ribose) polymerase in plants affects energy homeostasis, cell death and stress tolerance. Plant J 41: 95–106. 15610352 10.1111/j.1365-313X.2004.02277.x

[34] Vanderauwera S, De Block M, Van de Steene N, van de Cotte B, Metzlaff M, et al (2007) Silencing of poly(ADP-ribose) polymerase in plants alters abiotic stress signal transduction. Proc Natl Acad Sci U S A 104: 15150–15155. 17823244 10.1073/pnas.0706668104 PMC1986628

[35] Panda S, Poirier GG, Kay SA (2002) tej defines a role for poly(ADP-ribosyl)ation in establishing period length of the arabidopsis circadian oscillator. Dev Cell 3: 51–61. 12110167 10.1016/s1534-5807(02)00200-9

[36] Adams-Phillips L, Wan J, Tan X, Dunning FM, Meyers BC, et al (2008) Discovery of ADP-ribosylation and other plant defense pathway elements through expression profiling of four different Arabidopsis-Pseudomonas R-avr interactions. Mol Plant Microbe Interact 21: 646–657. 18393624 10.1094/MPMI-21-5-0646

[37] Adams-Phillips L, Briggs AG, Bent AF (2010) Disruption of Poly(ADP-ribosyl)ation Mechanisms Alters Responses of Arabidopsis to Biotic Stress. Plant Physiology 152: 267–280. 19889874 10.1104/pp.109.148049 PMC2799362

[38] Li GJ, Nasar V, Yang YX, Li W, Liu B, et al (2011) Arabidopsis poly(ADP-ribose) glycohydrolase 1 is required for drought, osmotic and oxidative stress responses. Plant Science 180: 283–291. 21421372 10.1016/j.plantsci.2010.09.002

[39] Asai T, Tena G, Plotnikova J, Willmann MR, Chiu WL, et al (2002) MAP kinase signalling cascade in Arabidopsis innate immunity. Nature 415: 977–983. 11875555 10.1038/415977a

[40] He P, Shan L, Lin NC, Martin GB, Kemmerling B, et al (2006) Specific bacterial suppressors of MAMP signaling upstream of MAPKKK in Arabidopsis innate immunity. Cell 125: 563–575. 16678099 10.1016/j.cell.2006.02.047

[41] Luna E, Pastor V, Robert J, Flors V, Mauch-Mani B, et al (2011) Callose deposition: a multifaceted plant defense response. Mol Plant Microbe Interact 24: 183–193. 20955078 10.1094/MPMI-07-10-0149

[42] Aravind L, Koonin EV (2000) SAP - a putative DNA-binding motif involved in chromosomal organization. Trends Biochem Sci 25: 112–114. 10694879 10.1016/s0968-0004(99)01537-6

[43] Le May N, Iltis I, Ame JC, Zhovmer A, Biard D, et al (2012) Poly (ADP-ribose) glycohydrolase regulates retinoic acid receptor-mediated gene expression. Mol Cell 48: 785–798. 23102699 10.1016/j.molcel.2012.09.021

[44] Krishnakumar R, Gamble MJ, Frizzell KM, Berrocal JG, Kininis M, et al (2008) Reciprocal binding of PARP-1 and histone H1 at promoters specifies transcriptional outcomes. Science 319: 819–821. 18258916 10.1126/science.1149250

[45] Krishnakumar R, Kraus WL (2010) PARP-1 regulates chromatin structure and transcription through a KDM5B-dependent pathway. Mol Cell 39: 736–749. 20832725 10.1016/j.molcel.2010.08.014 PMC2939044

[46] Wang Y, Li J, Hou S, Wang X, Li Y, et al (2010) A Pseudomonas syringae ADP-ribosyltransferase inhibits Arabidopsis mitogen-activated protein kinase kinases. Plant Cell 22: 2033–2044. 20571112 10.1105/tpc.110.075697 PMC2910962

[47] Fu ZQ, Guo M, Jeong BR, Tian F, Elthon TE, et al (2007) A type III effector ADP-ribosylates RNA-binding proteins and quells plant immunity. Nature 447: 284–288. 17450127 10.1038/nature05737

[48] de Murcia JMN, Ricoul M, Tartier L, Niedergang C, Huber A, et al (2003) Functional interaction between PARP-1 and PARP-2 in chromosome stability and embryonic development in mouse. Embo Journal 22: 2255–2263. 12727891 10.1093/emboj/cdg206 PMC156078

[49] Koh DW, Lawler AM, Poitras MF, Sasaki M, Wattler S, et al (2004) Failure to degrade poly(ADP-ribose) causes increased sensitivity to cytotoxicity and early embryonic lethality. Proc Natl Acad Sci U S A 101: 17699–17704. 15591342 10.1073/pnas.0406182101 PMC539714

[50] Hanai S, Kanai M, Ohashi S, Okamoto K, Yamada M, et al (2004) Loss of poly(ADP-ribose) glycohydrolase causes progressive neurodegeneration in Drosophila melanogaster. Proc Natl Acad Sci U S A 101: 82–86. 14676324 10.1073/pnas.2237114100 PMC314142

[51] Song J, Bent AF (2014) Microbial pathogens trigger host DNA double-strand breaks whose abundance is reduced by plant defense responses. PLoS Pathog 10: e1004030. 24699527 10.1371/journal.ppat.1004030 PMC3974866

[52] Toller IM, Neelsen KJ, Steger M, Hartung ML, Hottiger MO, et al (2011) Carcinogenic bacterial pathogen Helicobacter pylori triggers DNA double-strand breaks and a DNA damage response in its host cells. Proc Natl Acad Sci U S A 108: 14944–14949. 21896770 10.1073/pnas.1100959108 PMC3169107

[53] Yan S, Wang W, Marques J, Mohan R, Saleh A, et al (2013) Salicylic acid activates DNA damage responses to potentiate plant immunity. Mol Cell 52: 602–610. 24207055 10.1016/j.molcel.2013.09.019 PMC3863363

[54] Cohen-Armon M, Visochek L, Rozensal D, Kalal A, Geistrikh I, et al (2007) DNA-independent PARP-1 activation by phosphorylated ERK2 increases Elk1 activity: a link to histone acetylation. Mol Cell 25: 297–308. 17244536 10.1016/j.molcel.2006.12.012

[55] Kraus WL, Hottiger MO (2013) PARP-1 and gene regulation: progress and puzzles. Mol Aspects Med 34: 1109–1123. 23357755 10.1016/j.mam.2013.01.005

[56] He P, Shan L, Sheen J (2007) The use of protoplasts to study innate immune responses. Methods Mol Biol 354: 1–9. 17172739 10.1385/1-59259-966-4:1

[57] Kinkema M, Fan W, Dong X (2000) Nuclear localization of NPR1 is required for activation of PR gene expression. Plant Cell 12: 2339–2350. 11148282 10.1105/tpc.12.12.2339 PMC102222

