# Correction: Protein Poly(ADP-ribosyl)ation Regulates *Arabidopsis* Immune Gene Expression and Defense Responses

**DOI:** 10.1371/journal.pgen.1005294

**Published:** 2016-09-09

**Authors:** Baomin Feng, Chenglong Liu, Marcos V. V. de Oliveira, Aline C. Intorne, Bo Li, Kevin Babilonia, Gonçalo A. de Souza Filho, Libo Shan, Ping He

There is an error in the affiliation of author Gonçalo A. de Souza Filho. The correct affiliation is Center of Biosciences and Biotechnology, Darcy Ribeiro State University of Northern of Rio de Janeiro, Brazil
