# Supplementary figures and images for: Protein Poly(ADP-ribosyl)ation Regulates Arabidopsis Immune Gene Expression and Defense Responses
Source: PLoS Genet. 2015 Jan 8;11(1):e1004936. doi: 10.1371/journal.pgen.1004936 (PMC4287526; doi:10.1371/journal.pgen.1004936)

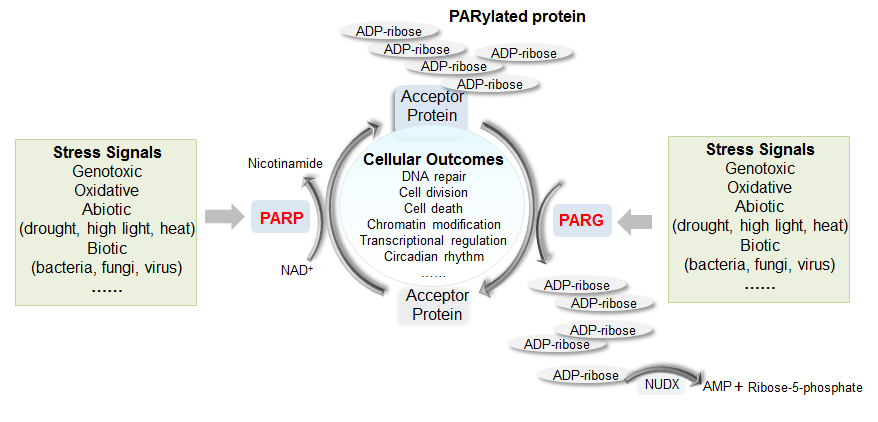

Supplement: S1 Fig — PARP- and PARG-mediated posttranslational PARylation in cellular stress responses. Extrinsic and intrinsic stress signals activate PARP which transfers ADP-ribose moiety from NAD+ to acceptor proteins resulting in the formation of linear or branched poly(ADP-ribose) (PAR) polymers. PARG could also be activated by different stresses and remove PAR polymers from acceptor proteins. Nucleoside diphosphate linked to some moiety-X (NUDX) then cleaves free ADP-ribose into AMP (adenosine monophosphate) and ribose-5-phosphate. (TIF) [file pgen.1004936.s001.tif]

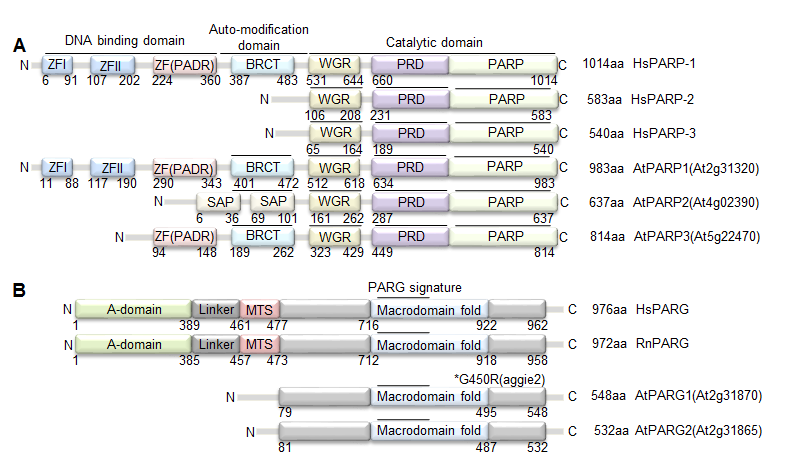

Supplement: S2 Fig — Domain organization of PARPs and PARGs. (A) Domain organization of human HsPARPs and Arabidopsis AtPARPs; ZFI: PARP-like zinc-finger1 domain; ZFII: PARP-like zinc-finger2 domain; ZF(PADR): zinc-binding domain 3; BRCT: BRCA1 carboxy-terminal domain for protein–protein and protein–DNA break binding domain; WGR: Trp-Gly-Arg in single letter code for putative PARP nucleic acid binding domain; PRD: PARP regulatory domain; PARP: PARP catalytic domain; SAP: SAF-A/B, Acinus and PIAS motif for putative DNA/RNA binding domain; (B) Domain structure of human HsPARG, Rat RnPARG and Arabidopsis AtPARGs. A-domain: N-terminal regulatory and targeting domain; MTS: mitochondrial targeting sequence; Macrodomain fold: core catalytic domain. The number under each domain indicates the position of amino acid in the protein. (TIF) [file pgen.1004936.s002.tif]

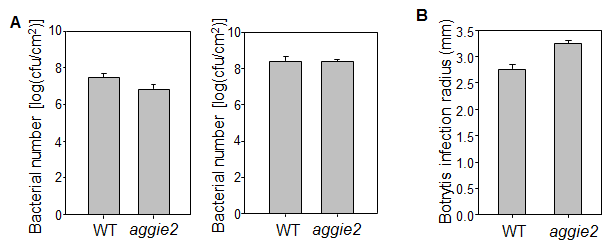

Supplement: S3 Fig — Disease assays in aggie2. (A) The aggie2 mutant response to Pst DC3000 infection. WT and aggie2 mutant plants were hand-inoculated with Pst DC3000 at OD600 = 5 × 10^-4^, and the bacterial counting was performed 3 days post-inoculation (dpi). The data are shown as mean ± se from three independent repeats. We performed 7 times of disease assays, and observed that aggie2 was more resistant than WT plants for 4 times, and there is no difference between aggie2 and WT for other 3 times. The representative bacterial counting with difference (left) or without difference (right) is shown. (B) The aggie2 mutant is more susceptible to B. cinerea infection. Leaves of six-week-old plants were drop-inoculated with B. cinerea at the concentration of 2.5 × 10^5^ spores/ml. Lesion size was measured 2 days post-inoculation. The data are shown as mean ± se from 20 infected leaves. (TIF) [file pgen.1004936.s003.tif]

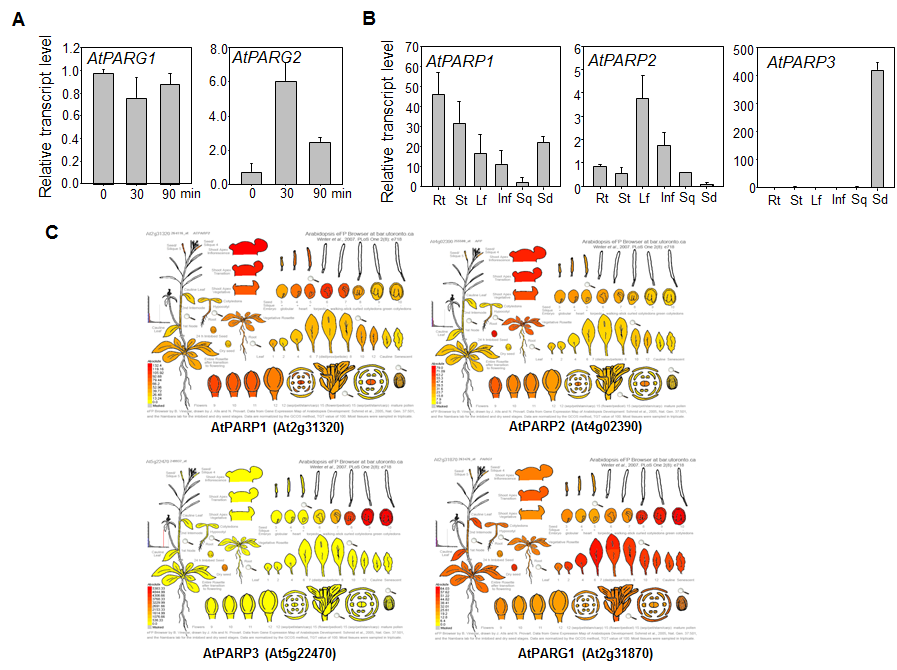

Supplement: S4 Fig — Expression pattern of AtPARG1, AtPARG2, AtPARP1, AtPARP2 and AtPARP3. (A) Response of AtPARG1 and AtPARG2 transcript level to flg22 treatment. The 12-day-old seedlings were treated with 100nM flg22 for qRT-PCR analysis. (B) The transcript levels of AtPARP1, AtPARP2 and AtPARP3 in 6 primary organs (Rt, root; St, Stem; Lf, leaf; Inf, inflorescence; Sq, silique; Sd, seeds) detected by qRT-PCR. AtPARP1 and AtPARP2 are expressed in all 6 organs. However, AtPARP3 is predominantly expressed in seeds but not in other organs (C) In silicon analysis of AtPARP1, 2, 3 and AtPARG1. The figures were obtained from Arabidopsis eFP Browser (http://bbc.botany.utoronto.ca/efp/cgi-bin/efpWeb.cgi) with indicated AGI numbers. Winter et al., 2007. PLoS One 2(8): e718. (TIF) [file pgen.1004936.s004.tif]

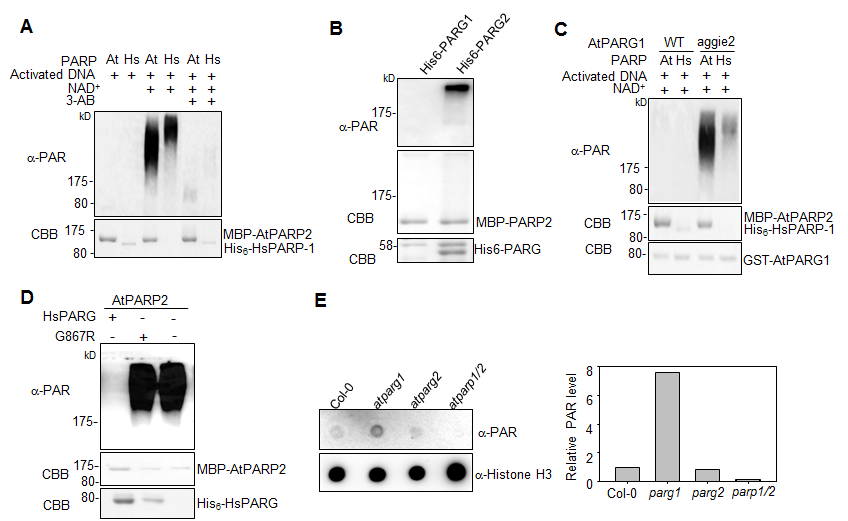

Supplement: S5 Fig — In vitro and in vivo activity of PARPs and PARGs. (A) The in vitro PARP activity of human HsPARP-1 and Arabidopsis AtPARP2 detected by an α-PAR Western blot. (B) In vitro enzymatic activity of His6-tagged AtPARG1 and AtPARG2. His6-AtPARG1, but not His6-AtPARG2, hydrolyzed PAR polymers from self-modified MBP-AtPARP2 shown as the disappearance of smear detected by α-PAR antibody. (C) GST-AtPARG1 hydrolyzes PAR polymers from self-modified AtPARP2 and HsPARP-1, and aggie2 mutation (G450R) blocks its activity. (D) HsPARGG867R, the corresponding mutation in aggie2, abolishes its PARG activity towards self-modified AtPARP2. (E). In vivo PAR level in Col-0, atparg1, atparg2 and atparp1/2. Nuclear protein extracts were isolated, dotted onto nitrocellulose membrane, probed with α-PAR antibody (left), and quantified with ImageJ software (right). Amount of nuclear proteins was normalized to the signal of α-Histone H3 antibody WB. The atparg1 mutant accumulates higher PAR polymers than Col-0; however, PAR polymer level in atparg2 is comparable with that in Col-0. (TIF) [file pgen.1004936.s005.tif]

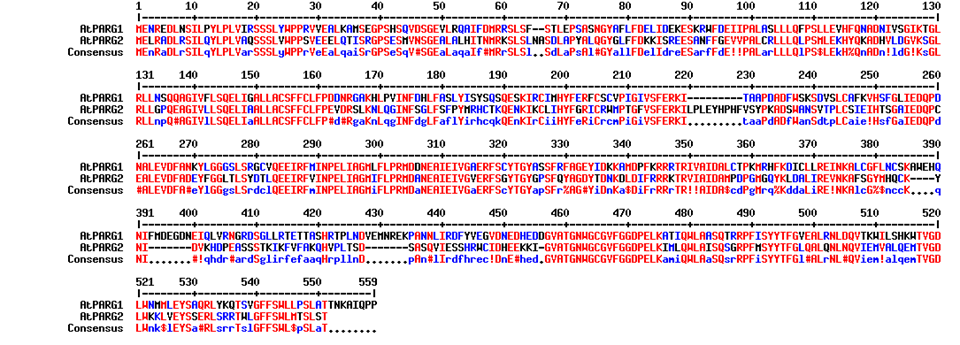

Supplement: S6 Fig — Comparison of AtPARG1 and AtPARG2 amino acid sequences. The alignment was generated with "Multiple sequence alignment with hierarchical clustering" F. CORPET, 1988, Nucl. Acids Res., 16 (22), 10881-10890. (http://multalin.toulouse.inra.fr/multalin/) (TIF) [file pgen.1004936.s006.tif]

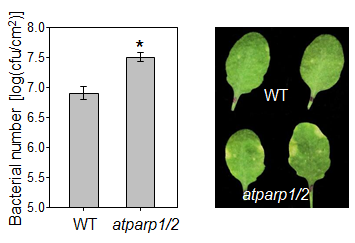

Supplement: S7 Fig — The atparp1/2 mutant is more susceptible to Pst DC3000 infection. WT and atparp1/2 double mutant plants were hand-inoculated with Pst DC3000 at OD600 = 5 × 10^-4^, and the bacterial counting was performed 3 days post-inoculation (dpi). The data are shown as mean ± se from three independent repeats with Student's t-test. * indicates p<0.05 when compared to WT (Left panel). The disease symptom is shown at 3 dpi (right panel). (TIF) [file pgen.1004936.s007.tif]

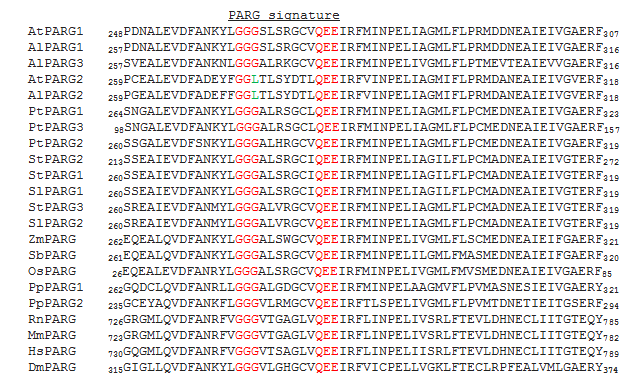

Supplement: S8 Fig — Sequence alignment of PARG signature motif among PARGs from different species. thale cress (Arabidopsis thaliana, At), lyrate rockcress (Arabidopsis lyrata, Al), poplar (Populus trichocarpa, Pt), potato (Solanum tuberosum, St), tomato (Solanum lycopersicum, Sl), maize(Zea mays, Zm), sorghum (Sorghum bicolor, Sb), rice (Oryza sativa, Os), moss(Physcomitrella patens, Pp), rat (Rattus norvegicus, Rn), mouse(Mus musculus, Mm), human (Homo sapiens, Hs), fruit fly (Drosophila melanogaster, Dm). The PARG signature motif is labeled in red. (TIF) [file pgen.1004936.s008.tif]
