# Supplementary material for: Protein Poly(ADP-ribosyl)ation Regulates Arabidopsis Immune Gene Expression and Defense Responses
Source: PLoS Genet. 2015 Jan 8;11(1):e1004936. doi: 10.1371/journal.pgen.1004936 (PMC4287526; doi:10.1371/journal.pgen.1004936)
Supplement: S1 Table — Cloning and point mutation primers (DOCX) [file pgen.1004936.s009.docx]

**Table S1**

Cloning and point mutation primers

| Gene | Forward primer | Reverse primer |
| --- | --- | --- |
| *AtPARG1* | TTTCC*ATG*GAGAATCGCGAAGATCTTAACTC | TTTAGGCCTTCG CGG CGGCTGGATAGCTTTG |
| *AtPARG2* | TTTGGATCC*ATG*GAACTGAGGGCAGATCTTAGGTC | CCCGATATCGGTAGACAGTGAGGTCATGAGCCAC |
| *AtPARP1* | CGCGGATCC*ATG*GCAAGCCCACATAAGCCGTG | GAAGGCCTTCTCTTGTGCTTAAACCTTAC |
| *AtPARP2* | TTTGGATCC*ATG*GCGAACAAGCTCAAAGTCGAC | ACTAGGCCTGTGCTTGTAGTTGAATTTGACTTGGATCAC |
| *Histone H1.1* | CGGGATCC*ATG*TCAGAGG TGGAAATAGAGAACG | GAAGGCCT CTTCTTAACC CTAGAAGAAGCCCTC |
| *pAtPARG1* | GGGGTACCCCTTTAATTAGAGAAAGTTTCTTCTTG | CGGAATTCCGCATGCCATGGCATGTTTTCGATTTTTCTAATCTCACAGC |
| *PARG1, E273A* | GTAGAGGGTGCGTGCAGGGAGAGATACGCTTCATGAT | ATCATGAAGCGTATCTCTCCCTGCACGCACCCTCTAC |
| *PARG1, F457A* | GAATTGGGGATGTGGTGTTGGTGGAGGAGACCCAGAGCT | AGCTCTGGGTCTCCTCCACCAACACCACATCCCCAATTC |
| *PARG1, G264L* | GCAAACAAGTATCTCGGAGGTCTTTCCCTAAGTAGAGGGTGCG | CGCACCCTCTACTTAGGGAAAGACCTCCGAGATACTTGTTTGC |
| *PARG2, L275G* | CAGATGAGTATTTTGGAGGCGGTACTCTGAGTTATGATACTC | GAGTATCATAACTCAGAGTACCGCCTCCAAAATACTCATCTG |
| *HsPARG, G867R* | CTTTCTGCAGTGGCCACACGAAACTGGGGCTGTGGTG | CACCACAGCCCCAGTTTCGTGTGGCCACTGCAGAAAG |

Note: The restriction enzyme sites are underlined and start codon is italicized; for point mutation primers, the mutated sites are underlined.
