# Supplementary material for: Protein Poly(ADP-ribosyl)ation Regulates Arabidopsis Immune Gene Expression and Defense Responses
Source: PLoS Genet. 2015 Jan 8;11(1):e1004936. doi: 10.1371/journal.pgen.1004936 (PMC4287526; doi:10.1371/journal.pgen.1004936)
Supplement: S2 Table — qRT-PCR primers (DOCX) [file pgen.1004936.s010.docx]

**Table S2**

qRT-PCR primers

| Gene | Forward primer | Reverse primer |
| --- | --- | --- |
| *FRK1* | ATCTTCGCTTGGAGCTTCTC | TGCAGCGCAAGGACTAGAG |
| *MYB15* | CTTGGCAATAGATGGTCAGC | TGAGTGTGCCATACGTTCTTG |
| *At2g17740* | TGCTCCATCTCTCTTTGTGC | ATGCGTTGCTGAAGAAGAGG |
| *UBQ10* | AGATCCAGGACAAGGAAGGTATTC | CGCAGGACCAAGTGAAGAGTAG |
